# Complete Genome Sequence and Comparative Genomic Analysis of *Mycobacterium massiliense* JCM 15300 in the *Mycobacterium abscessus* Group Reveal a Conserved Genomic Island MmGI-1 Related to Putative Lipid Metabolism

**DOI:** 10.1371/journal.pone.0114848

**Published:** 2014-12-11

**Authors:** Tsuyoshi Sekizuka, Masanori Kai, Kazue Nakanaga, Noboru Nakata, Yuko Kazumi, Shinji Maeda, Masahiko Makino, Yoshihiko Hoshino, Makoto Kuroda

**Affiliations:** 1 Pathogen Genomics Center, National Institute of Infectious Diseases, Tokyo, Japan; 2 Leprosy Research Center, National Institute of Infectious Diseases, Tokyo, Japan; 3 Research Institute of Tuberculosis, Japan Anti-Tuberculosis Association, Tokyo, Japan; Hopital Raymond Poincare - Universite Versailles St. Quentin, France

## Abstract

*Mycobacterium abscessus* group subsp., such as *M. massiliense, M. abscessus sensu stricto* and *M. bolletii*, are an environmental organism found in soil, water and other ecological niches, and have been isolated from respiratory tract infection, skin and soft tissue infection, postoperative infection of cosmetic surgery. To determine the unique genetic feature of *M. massiliense*, we sequenced the complete genome of *M. massiliense* type strain JCM 15300 (corresponding to CCUG 48898). Comparative genomic analysis was performed among *Mycobacterium* spp. and among *M. abscessus* group subspp., showing that additional ß-oxidation-related genes and, notably, the mammalian cell entry (*mce*) operon were located on a genomic island, *M. massiliense* Genomic Island 1 (MmGI-1), in *M. massiliense*. In addition, putative anaerobic respiration system-related genes and additional mycolic acid cyclopropane synthetase-related genes were found uniquely in *M. massiliense*. Japanese isolates of *M. massiliense* also frequently possess the MmGI-1 (14/44, approximately 32%) and three unique conserved regions (26/44; approximately 60%, 34/44; approximately 77% and 40/44; approximately 91%), as well as isolates of other countries (Malaysia, France, United Kingdom and United States). The well-conserved genomic island MmGI-1 may play an important role in high growth potential with additional lipid metabolism, extra factors for survival in the environment or synthesis of complex membrane-associated lipids. ORFs on MmGI-1 showed similarities to ORFs of phylogenetically distant *M. avium* complex (MAC), suggesting that horizontal gene transfer or genetic recombination events might have occurred within MmGI-1 among *M. massiliense* and MAC.

## Introduction

Nontuberculous mycobacteria (NTM) are classified into slowly growing mycobacterium (SGM) and rapidly growing mycobacterium (RGM) species; some of these bacteria cause pulmonary diseases [1]. Among RGM, the *Mycobacterium abscessus* group has been shown to be an emerging respiratory pathogen in cystic fibrosis, non-cystic-fibrosis bronchiectasis and chronic obstructive pulmonary disease [2], [3], [4], [5], [6], and is also an environmental organism found in soil, water and other ecological niches [7], [8]. The *M. abscessus* group consists of three subspecies, *M. abscessus* subsp. *abscessus* (*M. abscessus sensu stricto*), *M. abscessus* subsp. *massiliense* (*M. massiliense*) and *M. abscessus* subsp. *bolletii* (*M. bolletii*) [9], [10]. The three subspecies can generally be distinguished by phylogenetic analysis of the housekeeping gene, *rpoB*, and the macrolide resistance-related gene, erythromycin ribosome methyltransferase (*erm*) (41). Bryant *et al*. and Nakanaga *et al*. have recently reported more detailed classification methods, including, respectively, a whole-genome single nucleotide polymorphism (SNP) approach and a multiplex PCR method using insertion/deletion regions identified by whole-genome sequencing alignment analysis [4], [11]. Several subcutaneous infections following surgery, other medical treatments or traumatic injury have recently been found to be caused by *M. massiliense* [12], [13], [14], [15]. It was also recently reported that *M. massiliense* caused cutaneous infections that could not be attributed to a prior invasive procedure [16]. Phylogenetic analyses of the *M. abscessus* group have been performed, putative virulence factors of *M. abscessus sensu stricto* have been identified and studied, and the comparative whole-genome analysis of *M. abscessus* group isolated from patients of wide geographical origin have been performed [4], [17], [18], [19]; however, a detailed comparative analysis of *M. abscessus* group subspp. to determine *M. massiliense* unique genetic feature is lacking. Thus, in the current study, we sequenced the complete *M. massiliense* JCM 15300 (CCUG 48898) genome and compared it with that of *M. abscessus* group subspecies.

## Results and Discussion

### Genomic sequence of *M. massiliense* JCM 15300

**Figure 1:**
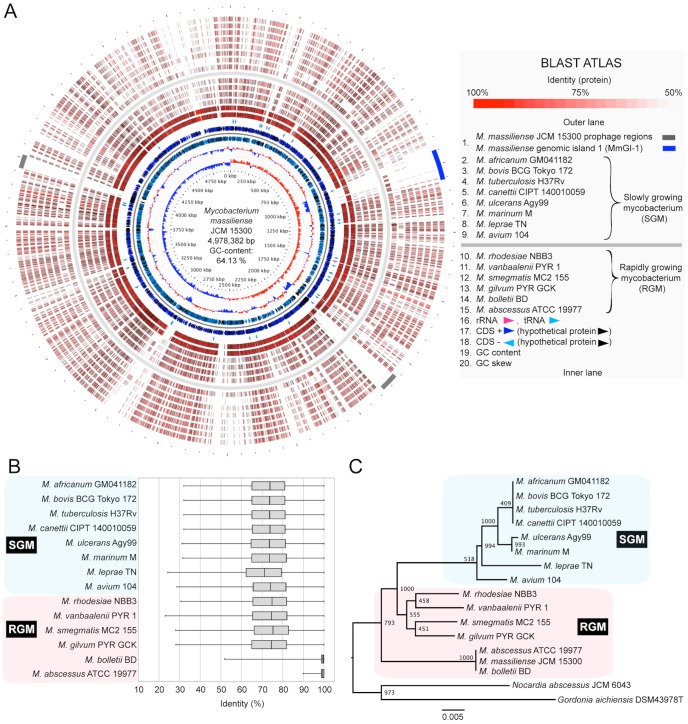
Circular representation of the *M. massiliense* JCM 15300 genome and comparative analysis among the complete genomes of *Mycobacterium* species. A. BLAST atlas of *M. massiliense* JCM 15300. The coding region of strain JCM 15300 was aligned against those of 14 other *Mycobacterium* genomes using BLASTP. The results are displayed as colored circles with increasing color intensity signifying increased similarity. It was estimated that the number of conserved proteins was 1,516 among all 14 *Mycobacterium* genomes. B. Box plot of identity percentage of conserved proteins between *M. massiliense* JCM 15300 and 14 other *Mycobacterium* spp. The top of each box in the box plot indicates the 75th percentile, the bottom of each box indicates the 25th percentile and the center bar represents the median. C. Neighbor-joining phylogenetic tree based on 16S rRNA gene sequencing of *Mycobacterium* with 1,000-fold bootstrapping. Scale bar indicates number of substitutions per site. The number at each branch node represents the bootstrapping value. *Nocardia abscessus* JCM 6043 (GenBank: AF430018) and *Gordonia aichiensis* DSM43978T (X80633) were used as outgroups.

The complete chromosomal sequence of *M. massiliense* JCM 15300 was obtained by *de novo* assembly of short reads followed by gap-closing using directed PCR. The genome consisted of 4,978,382 base pairs (bps) with a GC content of 64.1% and 4,950 predicted coding sequences (CDSs), 46 tRNA genes, one rRNA operon and two prophages (Fig. 1A). The chromosomal sequence corresponded to the predicted restriction fragment profiles obtained by PFGE analysis (data not shown). A draft genomic sequence of CCUG 48898 corresponding to JCM 15300 has been previously deposited in GenBank (NZ_AHAR01000000) by another research group. Thus, we performed a comparative pair-wise sequence alignment, revealing highly conserved synteny to the complete genomic sequence of JCM 15300 (S1 Figure and S1 Table). There were 188 mutations within 33 CDSs and 7 non-coding sites, suggesting that the differences between type strains may be due to frequent passaging and cultivation in various laboratories and bioresource centers. JCM15300 strain is smooth colony morphotype, and then there are no nonsense or frameshift mutations and in *mps1*-*mps2*-*gap* (MMASJCM_4183, MMASJCM_4184 and MMASJCM_4185) or *mmpl4b* (MMASJCM_4202) (data not shown), these data is consistent with a previous report [20].

### Comparative genomic analysis within the *Mycobacterium* genus

To characterize the genomic features of *M. massiliense* JCM 15300, a BLAST atlas analysis was performed; corresponding orthologs in complete and draft genomic sequences of other *Mycobacterium* spp. were compared with those of *M. massiliense* JCM 15300 as a reference (*M. bolletii* BD is a draft genomic sequence, but it is closely related to *M. massiliense*) (Fig. 1A). The BLAST atlas identified the conserved proteins in the core genome, which was represented by 973 CDSs (19.7%) shared among all 15 *Mycobacterium* spp. genomes. *M. massiliense* JCM 15300 was highly similar to *M. abscessus* ATCC 19977 and *M. bolletii* BD in the *M. abscessus* group (Fig. 1B). In contrast, *M. massiliense* JCM 15300 showed a low similarity (∼73% of mean identity) to SGM and other RGM (Fig. 1B). The 16S rRNA phylogenetic analysis suggested complete identity of *M. massiliense* JCM 15300 to *M. abscessus* ATCC 19977 and *M. bolletii* BD (Fig. 1C). These results indicate that *M. massiliense* is difficult to distinguish among the three *M. abscessus* subspecies using 16S rRNA gene phylogeny and that the three subspecies belong to the *M. abscessus* group as suggested by many reports.

The above analysis demonstrated that there were several highly variable gene clusters and notable differences in GC content (64.1%) among the 14 *Mycobacterium* spp. One prophage, located in the region from 1,816 to 1,880 kbs, had a lower GC content (59.64%) and partially shared some conserved CDSs with *M. abscessus* ATCC 19977 (gray bar in the lower right of Fig. 1A). The average GC content of all 14 *Mycobacterium* spp. and 620 mycobacteriophages [21] was approximately 66% and 64%, respectively, suggesting that the low-GC content prophage was recently acquired. In contrast, another prophage, located in the region from 3,964,186 to 4,013,302 bps, had an average GC content (64%), indicating that it could be specific to *M. massiliense* JCM 15300 (gray bar in the upper left of Fig. 1A).

**Figure 2:**
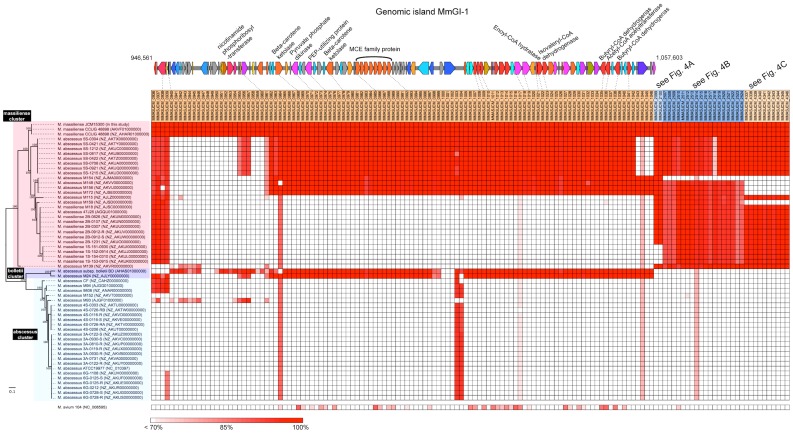
Schematic representation of genomic island MmGI-1 and heatmap of MmGI-1, anaerobic respiration genes and mycolic acid synthase-related gene loci among 56 *M. abscessus* group strains. Phylogenetic tree based on 203,267 core genome SNPs in the whole-genome-sequenced *M. abscessus* group by the maximum-likelihood method with 1,000-fold bootstrapping. The scale indicates that a branch with a length of 0.1 is 10 times as long as one that would show a 1% difference between the nucleotide sequences at the beginning and end of the branch. The number at each branch node represents the bootstrapping value. The ORFs of *M. massiliense* strain JCM 15300 were aligned against the genomic sequences of 56 other *M. abscessus* group strains and *M. avium* 104 using TBLASTN (E-value cutoff, 1.00E-10; identity cutoff, 70%). A heatmap was constructed from amino acid identity.

Intriguingly, a notable genomic island from 946,561 to 1,057,603 bps, designated *M. massiliense* genomic island 1 (MmGI-1; indicated by the blue bar in the upper right of Fig. 1A), appeared to be conserved among *M. massiliense* JCM 15300, *M. bolletii* BD and *M. avium* 104. The genomic island contained gene clusters associated with lipid metabolism and lipid-related transporters (Fig. 2 and Table 1). ß-oxidation-related genes were also identified, such as long-chain fatty acid-CoA ligase (MMASJCM_1018, MMASJCM_1019, MMASJCM_1028), acyl-CoA dehydrogenase (MMASJCM_1023, MMASJCM_1030, MMASJCM_1035, MMASJCM_1038), enoyl-CoA hydratase (MMASJCM_1008, MMASJCM_1009, MMASJCM_1010, MMASJCM_1022), 3-hydroxyacyl-CoA dehydrogenase (MMASJCM_1006, MMASJCM_1034), acyl-CoA thiolase (MMASJCM_1016, MMASJCM_1036) and acetyl-CoA acetyltransferase (MMASJCM_1014) (Table 1).

**Table 1:** Genes on the genomic island MmGI-1 *M. massiliense* JCM 15300.

Gene_ID	Location at JCM 15300	Strand	Length	Product	COG classifications*	KEGG orthology	BLASTP top hit seqeuence (E-value cutoff: 1E-1, database: nr without *M. abscessus* group data)			
							Accession number	Organisms	E-value	Identities
**MMASJCM_0936**	946561..947025	-	154	guanosine-3',5'-bis(Diphosphate) 3'-pyrophosphohydrolase	TK		WP_023955244.1	*Williamsia* sp. D3	7E-39	53.85%
**MMASJCM_0937**	947015..947167	-	50	hypothetical protein			WP_013871760.1	Frankia symbiont of Datisca glomerata	4E-06	47.73%
**MMASJCM_0938**	947284..949143	-	619	hypothetical protein	H		EUA75642.1	*M. chelonae* 1518	6E-161	69.98%
**MMASJCM_0939**	949143..949457	-	104	hypothetical protein	S		EUA75643.1	*M. chelonae* 1518	4E-22	54.74%
**MMASJCM_0940**	949859..950386	-	175	hypothetical protein			WP_015388818.1	*M. yongonense*	1E-72	66.27%
**MMASJCM_0941**	950404..951273	-	289	hypothetical protein	O		WP_023363492.1	*M. kansasii*	8E-67	49.62%
**MMASJCM_0942**	951280..952167	-	295	hypothetical protein	L		WP_023363490.1	*M. kansasii*	3E-118	62.93%
**MMASJCM_0943**	952344..952706	+	120	hypothetical protein	K		WP_015388820.1	*M. yongonense*	6E-37	68.42%
**MMASJCM_0944**	952851..953441	+	196	hypothetical protein			WP_015388821.1	*M. yongonense*	3E-54	61.96%
**MMASJCM_0945**	953484..954032	+	182	hypothetical protein			WP_015388822.1	*M. yongonense*	1E-69	58.56%
**MMASJCM_0946**	954019..955020	+	333	hypothetical protein			WP_015388823.1	*M. yongonense*	2E-154	72.50%
**MMASJCM_0947**	955027..955311	-	94	hypothetical protein	S		EWT07839.1	*Intrasporangium chromatireducens* Q5-1	2E-34	64.89%
**MMASJCM_0948**	956934..958430	-	498	site-specific DNA-methyltransferase	L		WP_020097565.1	*Microbacterium* sp. 11MF	7E-177	63.77%
**MMASJCM_0949**	958473..958796	+	107	hypothetical protein			WP_011768395.1	*Mycobacterium* sp. KMS	3E-08	36.56%
**MMASJCM_0950**	958893..959312	-	139	hypothetical protein			WP_006339348.1	*Gordonia rhizosphera*	1E-14	31.85%
**MMASJCM_0951**	959512..960780	+	422	hypothetical protein			WP_029121465.1	*Mycobacterium* sp. UNC410CL29Cvi84	1E-165	58.18%
**MMASJCM_0952**	960806..961159	+	117	hypothetical protein			WP_020099065.1	*Mycobacterium*	5E-36	58.49%
**MMASJCM_0953**	961156..961461	-	101	hypothetical protein	S		WP_024801663.1	*Nocardia* sp. BMG51109	2E-09	35.42%
**MMASJCM_0954**	961458..961751	-	97	hypothetical protein	S		WP_020099063.1	*Mycobacterium*	2E-19	48.45%
**MMASJCM_0955**	961838..962734	+	298	phosphoribosylpyrophosphate synthetase	FE		ETB46104.1	*M. avium* 10-5560	2E-48	51.56%
**MMASJCM_0956**	962749..964272	+	507	nicotinamide phosphoribosyltransferase	H	K03462	ETB46369.1	*M. avium* 10-5560	0	71.69%
**MMASJCM_0957**	964269..964919	+	216	possible DNA hydrolase	F	K03574	ETB46368.1	*M. avium* 10-5560	2E-66	53.00%
**MMASJCM_0958**	965195..965308	+	37	hypothetical protein			No hits found			
**MMASJCM_0959**	965479..965808	+	109	hypothetical protein	R		No hits found			
**MMASJCM_0960**	965980..967356	+	458	hypothetical protein	C		WP_024449466.1	*M. iranicum*	0	57.42%
**MMASJCM_0961**	967635..967844	-	69	hypothetical protein			WP_015388818.1	*M. yongonense*	9E-23	75.38%
**MMASJCM_0962**	968295..968783	-	162	hypothetical protein	S		WP_025089036.1	*Mycobacterium*	6E-47	50.00%
**MMASJCM_0963**	968949..969167	-	72	hypothetical protein			WP_015291571.1	*M. canettii*	5E-13	60.71%
**MMASJCM_0964**	969380..970636	-	418	putative cytochrome P450 IgrA	Q	K00517	EUA78264.1	*M. chelonae* 1518	0	88.04%
**MMASJCM_0965**	971395..971925	+	176	conserved hypothetical integral membrane protein YrbE1A	Q		WP_005143639.1	*M. rhodesiae*	1E-37	44.97%
**MMASJCM_0966**	971981..972526	-	181	transcriptional regulator, TetR family	K		WP_014384296.1	*M. intracellulare*	5E-53	50.00%
**MMASJCM_0967**	972591..973097	-	168	transcriptional regulator, TetR family	K		WP_014384297.1	*M. intracellulare*	2E-61	58.33%
**MMASJCM_0968**	973468..975162	+	564	beta-carotene ketolase	Q	K02292	CDO90343.1	*M. triplex*	0	91.41%
**MMASJCM_0969**	975672..976337	+	221	hypothetical protein	R		CDO30896.1	*M. vulneris*	5E-120	74.21%
**MMASJCM_0970**	976573..976902	+	109	hypothetical protein			WP_010228994.1	*Pseudonocardia* sp. P1	5E-27	52.88%
**MMASJCM_0971**	976927..978438	-	503	pyruvate, phosphate dikinase	G	K01006	WP_011726421.1	*M. avium*	0	72.06%
**MMASJCM_0972**	978435..979052	-	205	hypothetical protein	K		KDO99916.1	*M. avium* subsp. *hominissuis* 101	1E-95	67.80%
**MMASJCM_0973**	979096..980010	-	304	hypothetical protein			WP_011726419.1	*M. avium*	2E-177	79.28%
**MMASJCM_0974**	980007..981524	-	505	hypothetical protein	G	K01007	KBR61967.1	*M. tuberculosis* XTB13-223	0	73.76%
**MMASJCM_0975**	981770..982378	+	202	transcriptional regulator, TetR family	K		WP_011726417.1	*M. avium*	1E-85	66.67%
**MMASJCM_0976**	982618..983658	+	346	hypothetical protein			CDO30900.1	*M. vulneris*	0	87.32%
**MMASJCM_0977**	983932..984459	+	175	transcriptional regulator, TetR family	K		CDO90192.1	*M. triplex*	2E-61	60.00%
**MMASJCM_0978**	984571..986193	-	540	beta-carotene ketolase	Q		KDE98300.1	*M. aromaticivorans* JS19b1	0	82.45%
**MMASJCM_0979**	986685..987560	+	291	hypothetical protein			KDE98305.1	*M. aromaticivorans* JS19b1	2E-175	83.74%
**MMASJCM_0980**	987577..988209	-	210	transcriptional regulator, TetR family	K		KDE98304.1	*M. aromaticivorans* JS19b1	1E-95	76.60%
**MMASJCM_0981**	988316..989380	+	354	hypothetical protein			KDE98303.1	*M. aromaticivorans* JS19b1	0	77.68%
**MMASJCM_0982**	989396..990508	+	370	putative phosphotransferase	R		WP_005141265.1	*M. rhodesiae*	0	75.41%
**MMASJCM_0983**	990691..990807	+	38	hypothetical protein			No hits found			
**MMASJCM_0984**	990970..991083	-	37	hypothetical protein			No hits found			
**MMASJCM_0985**	991197..992228	+	343	putative YrbE family protein	Q		KBR61969.1	*M. tuberculosis* XTB13-223	2E-148	88.21%
**MMASJCM_0986**	992228..993097	+	289	putative Mce family protein	Q		KBR61970.1	*M. tuberculosis* XTB13-223	8E-168	80.28%
**MMASJCM_0987**	993105..994199	+	364	putative Mce family protein	Q		CDO30921.1	*M. vulneris*	0	70.56%
**MMASJCM_0988**	994196..995203	+	335	putative Mce family protein	Q		WP_011726414.1	*M. avium*	0	75.52%
**MMASJCM_0989**	995221..996162	+	313	putative Mce family protein	Q		KBR61973.1	*M. tuberculosis* XTB13-223	1E-176	77.96%
**MMASJCM_0990**	996132..997280	+	382	putative Mce family protein	Q		KDO99908.1	*M. avium* subsp. *hominissuis* 101	0	67.28%
**MMASJCM_0991**	997277..998266	+	329	putative Mce family protein	Q		WP_024637000.1	*M. avium*	2E-162	69.39%
**MMASJCM_0992**	998263..999219	+	318	putative Mce family protein	Q		CDO30926.1	*M. vulneris*	3E-157	69.50%
**MMASJCM_0993**	999262..999906	+	214	hypothetical protein			WP_007170571.1	*M. parascrofulaceum*	1E-82	61.27%
**MMASJCM_0994**	999982..1000584	+	200	hypothetical protein			KDE98251.1	*M. aromaticivorans* JS19b1	5E-88	65.83%
**MMASJCM_0995**	1000670..1001113	+	147	hypothetical protein			CDO30929.1	*M. vulneris*	7E-48	63.20%
**MMASJCM_0996**	1001158..1001496	+	112	hypothetical protein			WP_007170568.1	*M. parascrofulaceum*	4E-44	62.39%
**MMASJCM_0997**	1001544..1002104	+	186	hypothetical protein			CDO30931.1	*M. vulneris*	5E-91	75.71%
**MMASJCM_0998**	1002279..1002410	+	43	hypothetical protein			No hits found			
**MMASJCM_0999**	1002407..1003372	-	321	hypothetical protein	O		WP_014711294.1	*Mycobacterium* sp. MOTT36Y	0	80.94%
**MMASJCM_1000**	1003379..1004497	-	372	putative phosphotransferase	R		CDO90200.1	*M. triplex*	0	68.01%
**MMASJCM_1001**	1004938..1007496	-	852	hypothetical protein	K		WP_030203671.1	*Pilimelia anulata*	0	72.98%
**MMASJCM_1002**	1007489..1008457	-	322	cell division protein FtsH	O		WP_022566726.1	*Nocardia asteroides*	0	88.51%
**MMASJCM_1003**	1009865..1010737	+	290	hypothetical protein			EUA78068.1	*M. chelonae* 1518	4E-180	95.32%
**MMASJCM_1004**	1010796..1013315	+	839	hypothetical protein	D		WP_005113273.1	*M. chelonae*	0	94.89%
**MMASJCM_1005**	1015076..1015558	-	160	hypothetical protein	Q		WP_013873946.1	Frankia symbiont of Datisca glomerata	3E-23	45.45%
**MMASJCM_1006**	1015591..1016388	-	265	2-hydroxycyclohexanecarboxyl-CoA dehydrogenase	IQR		WP_011726451.1	*M. avium*	1E-162	83.77%
**MMASJCM_1007**	1016500..1017249	+	249	3-oxoacyl-[acyl-carrier protein] reductase	IQR	K00059	WP_023985895.1	*M. neoaurum*	2E-135	80.82%
**MMASJCM_1008**	1017246..1018016	+	256	enoyl-CoA hydratase	I	K15866	WP_011726449.1	*M. avium*	8E-104	66.54%
**MMASJCM_1009**	1018013..1018810	+	265	enoyl-CoA hydratase	I	K15866	WP_011726448.1	*M. avium*	4E-145	82.95%
**MMASJCM_1010**	1018810..1019595	+	261	enoyl-CoA hydratase	I	K15866	WP_029114372.1	*Mycobacterium* sp. URHB0044	7E-120	70.93%
**MMASJCM_1011**	1019592..1020860	+	422	putative dioxygenase hydroxylase component	PR	K05549	WP_030136631.1	*M. neoaurum*	0	86.46%
**MMASJCM_1012**	1021187..1021393	+	68	beta subunit of hydroxylase component of benzoate 1,2-dioxygenase	Q		WP_011726445.1	*M. avium*	3E-26	77.05%
**MMASJCM_1013**	1021459..1021659	+	66	hypothetical protein	T		WP_030136633.1	*M. neoaurum*	3E-29	81.54%
**MMASJCM_1014**	1021938..1022864	+	308	acetyl-CoA acetyltransferase	I	K00626	WP_014384231.1	*M. intracellulare*	0	84.36%
**MMASJCM_1015**	1022861..1024216	+	451	hydroxymethylglutaryl-CoA synthase	I		WP_011726442.1	*M. avium*	0	73.38%
**MMASJCM_1016**	1024206..1025411	+	401	putative thiolase	I		WP_011726441.1	*M. avium*	0	88.35%
**MMASJCM_1017**	1025490..1026350	+	286	probable short-chain type dehydrogenase reductase	IQR	K12405	WP_011726440.1	*M. avium*	4E-172	84.27%
**MMASJCM_1018**	1026409..1028046	+	545	long-chain-fatty-acid—CoA ligase	IQ	K01911	WP_011726439.1	*M. avium*	0	66.42%
**MMASJCM_1019**	1028043..1029800	+	585	long-chain-fatty-acid—CoA ligase	IQ		WP_011726438.1	*M. avium*	0	68.67%
**MMASJCM_1020**	1029761..1030786	-	341	hypothetical protein	R		WP_023985889.1	*M. neoaurum*	7E-128	57.19%
**MMASJCM_1021**	1030966..1031418	+	150	acyl dehydratase	I		WP_003923910.1	*M. thermoresistibile*	2E-76	75.00%
**MMASJCM_1022**	1031408..1032619	+	403	enoyl-CoA hydratase	I	K15866	WP_007170622.1	*M. parascrofulaceum*	2E-174	67.74%
**MMASJCM_1023**	1032620..1033783	+	387	isovaleryl-CoA dehydrogenase	I		WP_007170621.1	*M. parascrofulaceum*	0	81.61%
**MMASJCM_1024**	1033815..1035116	+	433	phytoene dehydrogenase family protein	Q		WP_007170620.1	*M. parascrofulaceum*	0	81.73%
**MMASJCM_1025**	1035104..1035961	+	285	citrate lyase beta chain	G	K01644	WP_007170619.1	*M. parascrofulaceum*	9E-111	66.92%
**MMASJCM_1026**	1036061..1036291	-	76	hypothetical protein			No hits found			
**MMASJCM_1027**	1036800..1037204	+	134	hypothetical protein	I		CDO90349.1	*M. triplex*	4E-79	88.06%
**MMASJCM_1028**	1037208..1038746	+	512	long-chain-fatty-acid—CoA ligase	IQ	K00666	WP_030136653.1	*M. neoaurum*	0	76.32%
**MMASJCM_1029**	1038743..1040002	+	419	putative cytochrome P450 hydroxylase	Q	K00517	CDO30946.1	*M. vulneris*	0	90.31%
**MMASJCM_1030**	1040014..1040805	+	263	3-alpha-hydroxysteroid dehydrogenase	IQR		WP_019509868.1	*M. neoaurum*	9E-156	82.89%
**MMASJCM_1031**	1040815..1042215	+	466	aldehyde dehydrogenase	C	K00128	WP_003923898.1	*M. thermoresistibile*	0	75.28%
**MMASJCM_1032**	1042215..1042406	+	63	hypothetical protein	C		WP_005141491.1	*M. rhodesiae*	3E-19	66.13%
**MMASJCM_1033**	1042569..1044056	+	495	ferredoxin—NADP(+) reductase	ER	K00528	KBR61952.1	*M. tuberculosis* XTB13-223	0	64.02%
**MMASJCM_1034**	1044016..1045248	+	410	4-hydroxybutyrate coenzyme A transferase	C		WP_011726433.1	*M. avium*	0	69.07%
**MMASJCM_1035**	1045317..1046471	-	384	butyryl-CoA dehydrogenase	I		WP_019509874.1	*M. neoaurum*	0	84.03%
**MMASJCM_1036**	1046475..1047626	-	383	acetyl-CoA acetyltransferase	I	K07823	WP_011726431.1	*M. avium*	0	87.21%
**MMASJCM_1037**	1047688..1048263	-	191	transcriptional regulator, TetR family	K		WP_030136662.1	*M. neoaurum*	6E-93	71.96%
**MMASJCM_1038**	1048446..1049600	-	384	butyryl-CoA dehydrogenase	I	K00248	WP_014941082.1	*M. indicus pranii*	0	84.38%
**MMASJCM_1039**	1049725..1050264	-	179	transcriptional regulator, TetR family	K		WP_019509888.1	*M. neoaurum*	3E-67	60.12%
**MMASJCM_1040**	1050416..1051048	-	210	transcriptional regulator, TetR family	K		WP_005146732.1	*M. rhodesiae*	6E-102	74.00%
**MMASJCM_1041**	1051285..1052259	+	324	hypothetical protein	I		WP_003938179.1	*Rhodococcus ruber*	5E-121	60.67%
**MMASJCM_1042**	1052411..1053019	+	202	transcriptional regulator, TetR family protein, putative	K		WP_014384219.1	*M. intracellulare*	5E-97	71.14%
**MMASJCM_1043**	1053327..1053584	+	85	hypothetical protein			WP_005111625.1	*M. chelonae*	2E-21	58.54%
**MMASJCM_1044**	1053701..1055929	+	742	carbonic anhydrase	P	K01673	WP_005057131.1	*M. chelonae*	0	76.16%
**MMASJCM_1045**	1056430..1056960	+	176	hypothetical protein			WP_028655880.1	*Nocardioides* sp. J54	2E-11	32.62%
**MMASJCM_1046**	1057007..1057603	+	198	hypothetical protein	G		WP_003960345.1	*Streptomyces clavuligerus*	2E-05	37.18%

*COG codes is as follows: C: Energy production and conversion, D: Cell cycle control, cell division, chromosome partitioning, E: Amino acid transport and metabolism, F: Nucleotide transport and metabolism, G: Carbohydrate transport and metabolism, H: Coenzyme transport and metabolism, I: Lipid transport and metabolism, K: Transcription, L: Replication, recombination and repair, O: Posttranslational modification, protein turnover, chaperones, P: Inorganic ion transport and metabolism, Q: Secondary metabolites biosynthesis, transport and catabolism, R: General function prediction only, S: Function unknown, T: Signal transduction mechanisms.

An ortholog of the mammalian cell entry (*mce*) operon (MMASJCM_0985 to _0992) was found in the genomic island (Fig. 2 and Table 1). The *mce* operon of *Actinomycetales* species has been suggested to encode a subfamily of ATP-binding cassette (ABC) transporters that have a possible role in remodeling the cell envelope [22] and entry of the pathogen into non-phagocytic cells [23]. Although the function of the Mce protein family has not been clearly established, its members are believed to be membrane lipid transporters. For example, it has been demonstrated that the *mce4* operon is required for cholesterol utilization and uptake by *M. tuberculosis* [24] and *M. smegmatis* [25]. *M. massiliense* JCM 15300 contained 8 loci from the *mce* operon, and one *mce* operon on the MmGI-1 genomic island demonstrated approximately 99% similarity to that of *M. bolletii* BD and approximately 80% similarity to that of *M. avium* 104.

**Figure 3:**
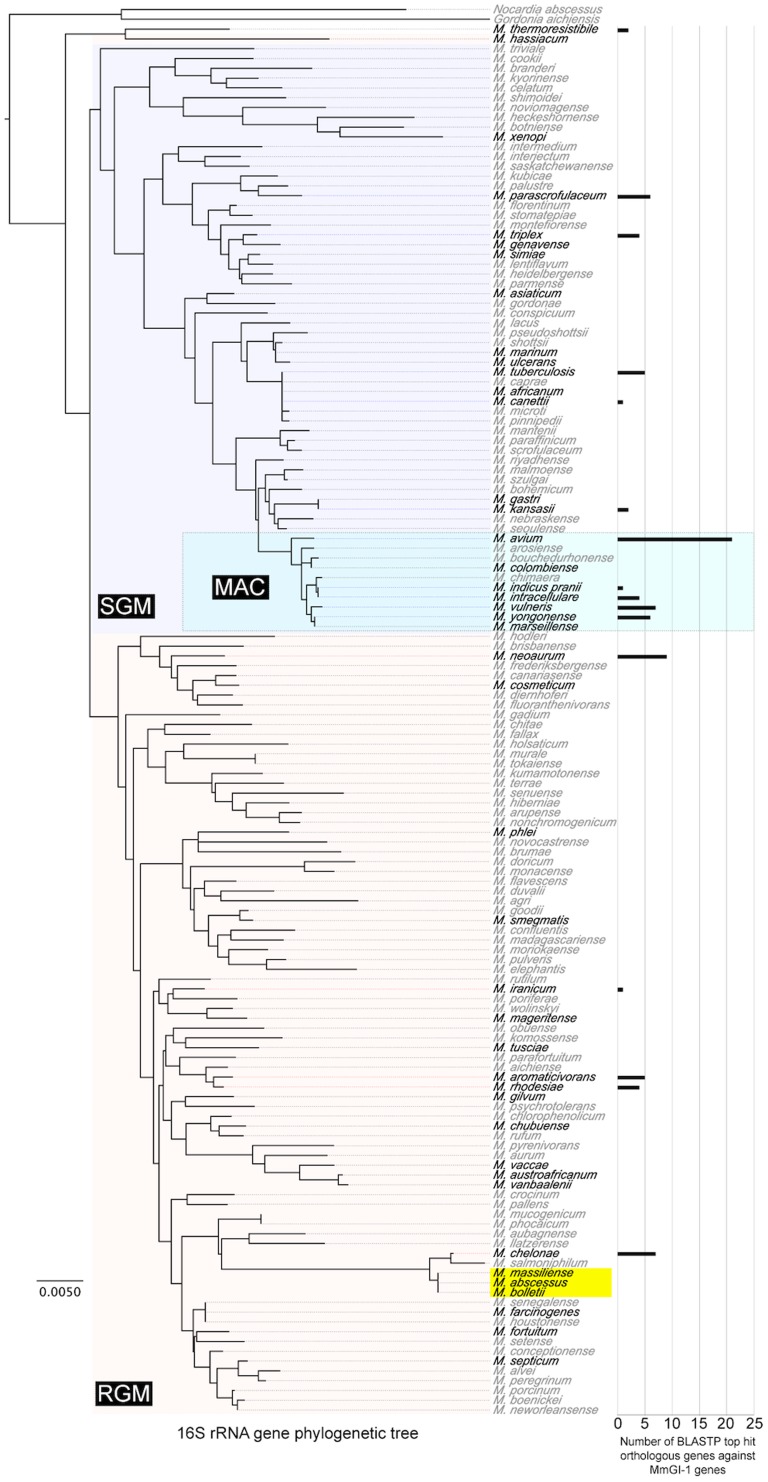
Orthologous genes of MmGI-1 genes in *Mycobacterium* spp. without *M. abscessus* group. Phylogenetic tree based on the 16S rRNA was constructed by Neighbor-joining method with 1,000-fold bootstrapping. Scale bar indicates number of substitutions per site. Species of black characters indicate that complete or draft genome sequences have been deposited at DDBJ/EMBL/GenBank. *M. abscessus* group is labeled by a yellow box. The number of BLASTP top hit orthologous genes against MmGI-1 genes are shown with a right bar chart.

To characterize a provenance of MmGI-1 regions, the regions were subjected to BLASTN/BLASTP search against NCBI nt/nr databases excluding *M. abscesses* group sequences. Although the nucleotide search with BLASTN did not show notable homology to MmGI-1 region, the protein search with BLASTP showed that 105 ORFs on MmGI-1 showed significant similarity to ORFs of *Actinomycetales* with 32 to 95% identity. Of 105 ORFs, forty-two ORFs showed similarities to ORFs of phylogenetically distant *M. avium* complex (MAC) (Fig. 3), suggesting that the MmGI-1 region might have been acquired through horizontal gene transfer or genetic recombination events with MAC.

Using 55 draft genomic sequences from the *M. abscessus* group [17] and one complete genomic sequence from *M. massiliense* JCM 15300, variation among the genomic islands was investigated. The phylogeny of *M. abscessus* group strains was further characterized by identifying 203,267 SNPs in the commonly shared genomic sequence (Fig. 2). The SNP phylogenetic analysis identified three clusters (i.e., massiliense, bolletii and abscessus clusters) from the *M. abscessus* group, consistent with a previous report [17]. Phylogenetic and heatmap analyses suggested that MmGI-1 was partially shared among *M. massiliense*-related strains (Fig. 2). Notably, the ß-oxidation-related loci (MMASJCM_0982 to _1042) were also well conserved in *M. bolletii* BD and M24. These additional lipid-related metabolic genes may be important for high growth potential with additional lipid metabolism such as putative ß-oxidation pathway, extra factors for survival in the environment (as suggested by the presence of MCE family protein) or synthesis of complex membrane-associated lipids (as suggested by the presence of a long-chain-fatty-acid-CoA ligase).

### Comparative genomic analysis within the *M. abscessus* group

To characterize the genomes of the previously described three clusters, we performed further comparative and BLAST atlas analyses based on the nucleotide sequences of two complete genomes and the predicted amino acid sequences of CDSs, respectively (S2 Figure and S2 and S3 Table), and then also performed pan-genomic analysis with 30 *M. massiliense*, 2 *M, bolletii* and 25 *M. abscessus* genome sequences because of a validation (S3 Figure). The pan-genomic analysis data is consistent with a previous report [19]. The comparative analysis yielded the following four results: i) as a massiliense cluster-specific feature, there were six unique regions (†^1–6^ in S2 Figure and Table 2) that contained an average GC content of 64%; ii) as a JCM 15300-specific feature, there were 10 unique regions (• in S2 Figure and S2 Table) that had relatively low GC content; iii) the MmGI-1 genomic island (Fig. 3 and ¶ in S2 Figure) was shared with *M. bolletii* and showed partial similarity to *M. avium* 104; iv) there were two common deletions (†^7–8^ in S2 Figure and S3 Table) in the massiliense cluster and one conserved region in the abscessus group (§ in S2 Figure and S3 Table).

**Table 2:** The unique conserved gene loci in massiliense cluster among *M. abscessus* group.

Gene_ID	Location at JCM 15300	Strand	Length	Product	Note
**MMASJCM_0834**	825792..826802	-	336	transcriptional regulator	
**MMASJCM_0835**	826913..827713	+	266	short chain dehydrogenase	
**MMASJCM_2099**	2098058..2101435	-	1125	putative molybdopterin oxidoreductase	see Fig. 4A
**MMASJCM_2100**	2101513..2102112	+	199	putative transcriptional regulator	see Fig. 4A
**MMASJCM_2410**	2427416..2427601	-	61	hypothetical protein	
**MMASJCM_2411**	2427632..2428042	+	136	hypothetical protein	
**MMASJCM_2412**	2428054..2428788	+	244	hypothetical protein	
**MMASJCM_2507**	2509971..2510735	-	254	universal stress protein family	see Fig. 4B
**MMASJCM_2508**	2510875..2511216	-	113	universal stress protein family	see Fig. 4B
**MMASJCM_2509**	2511996..2512505	+	169	probable conserved transmembrane protein	see Fig. 4B
**MMASJCM_2510**	2512542..2513558	+	338	alcohol dehydrogenase	see Fig. 4B
**MMASJCM_2511**	2513572..2514579	-	335	hypothetical protein	see Fig. 4B
**MMASJCM_2512**	2514754..2515698	+	314	universal stress protein family	see Fig. 4B
**MMASJCM_2513**	2515695..2518106	+	803	xylulose-5-phosphate phosphoketolase	see Fig. 4B
**MMASJCM_2514**	2518103..2518852	+	249	two component transcriptional regulatory protein DevR	see Fig. 4B
**MMASJCM_2515**	2518819..2519823	+	334	sensor kinase	see Fig. 4B
**MMASJCM_2516**	2519946..2520536	+	196	histidine kinase response regulator	see Fig. 4B
**MMASJCM_2517**	2520544..2521497	+	317	sulfate transporter	see Fig. 4B
**MMASJCM_2518**	2521466..2522251	+	261	sulfate transporter	see Fig. 4B
**MMASJCM_2519**	2522241..2522855	-	204	hypothetical protein	see Fig. 4B
**MMASJCM_2520**	2522957..2523163	-	68	hypothetical protein	see Fig. 4B
**MMASJCM_2521**	2523183..2524058	-	291	universal stress protein family	see Fig. 4B
**MMASJCM_2522**	2524296..2525168	+	290	universal stress protein family	see Fig. 4B
**MMASJCM_2523**	2525188..2525475	+	95	hypothetical protein	see Fig. 4B
**MMASJCM_2524**	2525508..2525942	+	144	hypothetical protein	see Fig. 4B
**MMASJCM_2869**	2886124..2887602	+	492	carotenoid oxygenase	
**MMASJCM_2870**	2887612..2888793	+	393	two-component system	
**MMASJCM_2871**	2888790..2889410	+	206	two component transcriptional regulator	
**MMASJCM_2872**	2890468..2892372	-	634	hypothetical protein	
**MMASJCM_2989**	3016494..3018116	+	540	diaminopimelate decarboxylase	
**MMASJCM_3589**	3593912..3594541	-	209	transcriptional regulator	
**MMASJCM_3590**	3594814..3595809	+	331	2-amino-3-carboxymuconate-6-semialdehyde decarboxylase	
**MMASJCM_4337**	4335727..4337094	-	455	deoxyribodipyrimidine photolyase	see Fig. 4C
**MMASJCM_4338**	4337091..4338449	-	452	cell division inhibitor	see Fig. 4C
**MMASJCM_4339**	4338477..4339142	-	221	hypothetical protein	see Fig. 4C
**MMASJCM_4340**	4339165..4340058	-	297	cyclopropane-fatty-acyl-phospholipid synthase	see Fig. 4C
**MMASJCM_4341**	4340280..4341596	+	438	amine oxidase	see Fig. 4C
**MMASJCM_4342**	4341593..4342330	+	245	hypothetical protein	see Fig. 4C
**MMASJCM_4343**	4342327..4343601	+	424	S-adenosyl-L-methionine dependent methyltransferase	see Fig. 4C
**MMASJCM_4344**	4343598..4344383	+	261	hypothetical protein	see Fig. 4C
**MMASJCM_4345**	4344416..4344961	+	181	RNA polymerase sigma-70 factor	see Fig. 4C
**MMASJCM_4346**	4344943..4345665	+	240	hypothetical protein	see Fig. 4C

**Figure 4:**
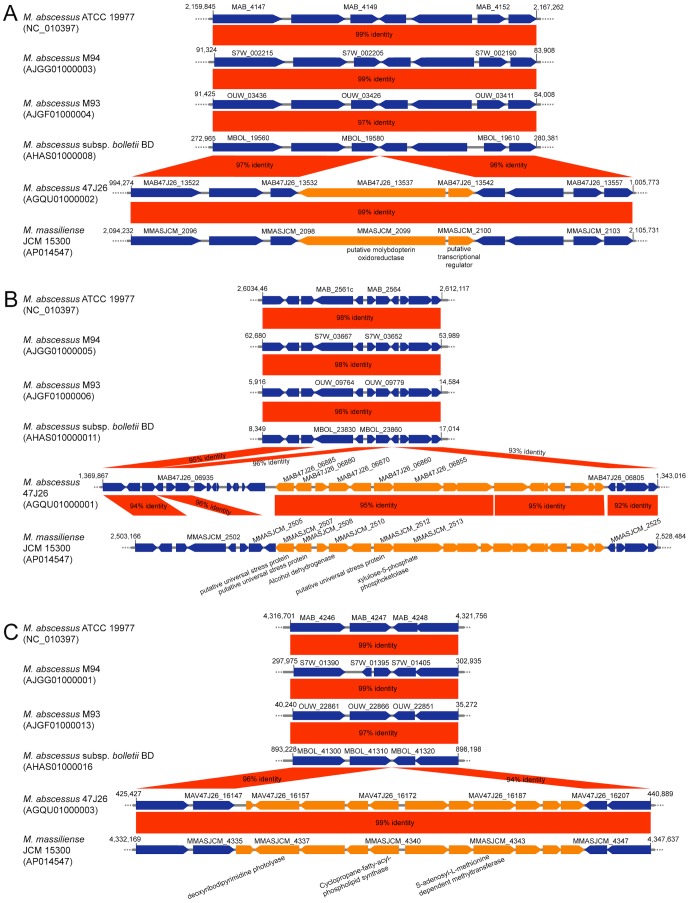
Comparison of unique genes and flanking regions in the massiliense cluster. GenBank accession numbers are given in parentheses. The orange arrows indicate the unique genes in the massiliense cluster. BLASTN match scores less than 200 are not shown.

In addition to the MmGI-1 genomic island described above, the massiliense cluster contained three notable conserved loci: i) a molybdopterin oxidoreductase (Fig. 2, Fig. 4A and Table 2); ii) universal stress proteins, an alcohol dehydrogenase and a xylulose-5-phosphate phosphoketolase (Fig. 2, Fig. 4B and Table 2); iii) a cyclopropane fatty acyl-phospholipid synthase and an S-adenosyl-L-methionine-dependent methyltransferase (Fig. 2, Fig. 4C and Table 2). In contrast to MmGI-1, these three regions were well conserved within the massiliense cluster.

Choo *et al*. previously reported that a high proportion of accessory strain-specific genes indicating an open, non-conservative pan-genome structure, and clear evidence of rapid phage-mediated evolution [19]. In fact, specific genes in *M. massiliense* JCM15300 contained phage-related genes, i.e. putative prophage integrase (S2 Table). On the other hand, in adjacent gene loci of three conserved regions, i.e. MMASJCM-2099..2100, MMASJCM-2507..2524 and MMASJCM-4337..4346, there are no phage-related genes (Fig. 4 and Table 2). These data suggest that these conserved regions might be core-genome regions in ancestral *M. abscessus* group, and then have been deleted from genomes of *M. abscessus* and *M. bolletii*.

### Prevalence of MmGI-1 and massiliense cluster unique regions in Japanese *M. massiliense* and *M. abscessus* isolates

**Figure 5:**
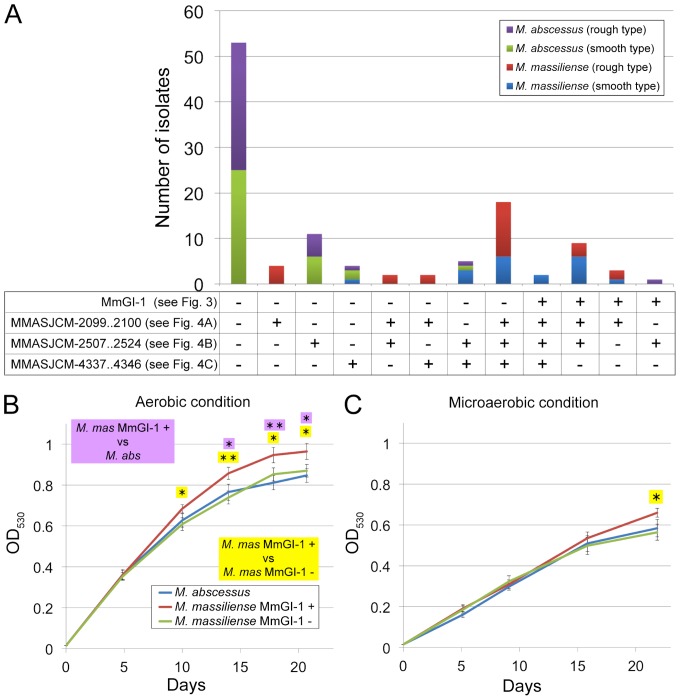
Prevalence of massiliense cluster unique regions and growth curve analysis in Japanese *M. massiliense* and *M. abscessus* isolates. A bar chart showing the prevalence of MmGI-1 and three massiliense cluster unique regions in Japanese *M. massiliense* and *M. abscessus* isolates (A). The curves represent *in vitro* growth (OD at 530 nm) over a period of 21 days at 37°C in aerobic (B) and microaerobic (C) conditions. Data represent the means ± SE from 6 MmGI-1 positive *M. massiliense*, 8 MmGI-1 negative *M. massiliense* and 12 *M. abscessus* isolates. *M. mas* and *M. abs* shows *M. massiliense* and *M. abscessus*, respectively. Key: +, positive; -, negative. * *P*<0.05; ** *P*<0.01 (Student's t-test).

We examined the prevalence of MmGI-1 and three massiliense cluster unique regions in Japanese *M. massiliense* and *M. abscessus* isolates using conventional PCR methods (S4 Table), because of *in silico* analysis using only isolates of Malaysia, France, United Kingdom and United States. The ratio of MmGI-1 positive *M. massiliense* and *M. abscessus* was 31.8% (14/44) and 1.4% (1/70), respectively (Fig. 5A and S5 Table). Applying Fisher's exact test, the proportion of MmGI-1 positive *M. massiliense* is significantly higher than that of *M. abscessus* (*P* = 0.0001). *M. massiliense* frequently possesses three massiliense cluster unique regions in not only Japanese but also other countries (Malaysia, France and United States) isolates (Fig. 5A and S5 Table), suggesting that MmGI-1 and the massiliense cluster unique regions are highly conserved in *M. massiliense* isolated from various countries.

### Growth ability of MmGI-1 positive *M. massiliense*

The massiliense cluster contained a conserved molybdopterin oxidoreductase as described above, and an ortholog was also identified in the strictly anaerobic bacterium, *Desulfitobacterium hafniense*. It has been reported that molybdopterin oxidoreductase may provide the ability for anaerobic energy metabolism [26]. The xylulose-5-phosphate phosphoketolase may play a role in heterolactic fermentation in anaerobic heterolactic acid bacteria, including *Lactobacillus* and *Leuconostoc* organisms [27]. Moreover, the universal stress protein in *Pseudomonas aeruginosa* has been reported to have a crucial role in survival under anaerobic conditions [28]. These studies suggest that *M. massiliense* may grow or survive under anaerobic or hypoxic conditions. Indeed, the oxygen partial pressure in various tissues is approximately 20–50 mm Hg (3–7% oxygen) [29], [30], [31], [32]. To determine growth ability under hypoxic conditions, 27 smooth colony morphology isolates (12 *M. abscessus*, 8 MmGI-1 positive *M. massiliense* and 7 MmGI-1 negative *M. massiliense* isolates) were subjected to aerobic and microaerobic (approximately 6% O_2_) conditions (Fig. 5B and 5C), because the aggregation of rough colony morphology isolates were hard to measure the degree of turbidity in the broth culture. In aerobic condition, MmGI-1 positive *M. massiliense* isolates show well growth than MmGI-1 negative isolates including *M. abscessus* (Fig. 5B). On the other hand, in microaerobic condition, the growth didn't show significant differences between *M. massiliense* and *M. abscessus* (Fig. 5C). MMASJCM-2099..2100 and MMASJCM-2057..2524 regions highly conserved in *M. massiliense* isolated from Japan, Malaysia, France, United Kingdom and United States, as well as MmGI-1. Although functions of these regions are still unclear, the importance of MmGI-1 might be supported by the existence on these conserved regions in *M. massiliense*, and MmGI-1 might relate to high growth potential with additional lipid metabolism such as putative ß-oxidation pathway.

### Phylogenetic analysis of mycolic acid synthase-related genes

The comparative genomic analysis indicated that *M. massiliense* including Japanese isolates possessed two extra CDSs that are possibly involved in the cyclopropanation of mycolic acid. A cyclopropane fatty acyl-phospholipid synthase (MMASJCM_4340) and an S-adenosyl-L-methionine-dependent methyltransferase (MMASJCM_4343) were detected only in the massiliense cluster (Fig. 4C). Both putative proteins encoded by these CDSs possessed the mycolic acid cyclopropane synthetase (CMAS) domain (pfam02353). *Mycobacterium* spp. possess 3 to 10 paralogs with a CMAS domain; for example, CmaA (cyclopropane mycolic acid synthase) and MmaA (methyl mycolic acid synthase) have been well characterized [33]. A phylogenetic analysis of CMAS domain-related proteins has indicated that one of the two extra proteins, MMASJCM_4340, is orthologous to MSMEG_1351 of *M. smegmatis* and MycrhN_0769/MycrhN_3064 of *M. rhodesiae* (S4 Figure). The other protein, MMASJCM_4343, is orthologous to UfaA1 (cyclopropane fatty acid synthase), which is present in a part of RGM and SGM species. The function of UfaA1 in mycolate biosynthesis is not clear [34]. The massiliense cluster has two unique mycolic acid synthesis-associated proteins that are not present in the abscessus or bolletii clusters.

## Conclusions

The *M. abscessus* group is classified as RGM species and consists of three closely related organisms, *M. abscessus, M. bolletii* and *M. massiliense*. A comparative analysis based on three clusters in the *M. abscessus* group revealed that a genomic island MmGI-1 of *M. massiliense* may be involved in high growth potential with additional lipid metabolism such as putative ß-oxidation pathway. Moreover, MmGI-1 is conserved in *Actinomycetales*, especially *Mycobacterium*, and horizontal gene transfer or genetic recombination events might have occurred within MmGI-1 among *M. massiliense* and MAC. Although *M. abscessus* subspp. is an environmental organism found in soil, water and other ecological niches, the difference of detail ecological niches is still unclear among subspecies-level. Our data suggests that the massiliense cluster unique regions including MmGI-1 might be linked to differences in ecological niches, such as lipid rich environment, of *M. massiliense* and *M. abscessus*. Further studies are required to understand the specific genetic features identified in this study.

## Materials and Methods

### Bacterial strains

We sequenced *Mycobacterium massiliense* type strain JCM 15300 (CCUG 48898), which was originally isolated from the sputum of a 50-year-old woman with an 8-year history of bronchiectasis and hemoptysis [35]. This strain was obtained from the Japan Collection of Microorganisms at the Riken BioResource Center (BRC-JCM; Saitama, Japan) on September 18, 2009.

### Short-read DNA sequencing

An *M. massiliense* strain DNA library (insert size of ∼600 bp) was prepared using the Nextera DNA Sample Prep Kit (Illumina-compatible) (EPICENTRE Biotechnologies, Madison, WI). DNA clusters were generated on a slide using the Cluster Generation Kit (ver. 4) on an Illumina Cluster Station (Illumina, San Diego, CA), according to the manufacturer's instructions. A paired-end sequencing run for 83 mers was performed using an Illumina Genome Analyzer IIx (GA IIx) with the TruSeq SBS Kit v5. Fluorescent images were analyzed using the Illumina RTA1.8/SCS2.8 base-calling pipeline to obtain FASTQ-formatted sequence data.

### *De novo* assembly of short DNA reads and gap-closing

Prior to *de novo* assembly, the obtained 80-mer reads were assembled using ABySS-pe v1.2.5 [36] with the following parameters: k60, n60, c68.4, t10, e10 and q20. Predicted gaps were amplified with specific PCR primer pairs followed by Sanger DNA sequencing with the BigDye Terminator v3.1 Cycle Sequencing Kit (Applied Biosystems, Foster City, CA).

### Validation of gap closing and sequencing errors by short-read mapping

To determine whether mis-assembled sequences and incorrect gap-closing remained after reference-assisted gap-closing, 40-mer short reads were aligned to the tentative complete chromosomal DNA sequence using Maq software (ver. 0.7.1) with the easyrun Perl command [37]. We then performed a read alignment to validate possible errors using the MapView graphical alignment viewer [38].

### Annotation

Gene prediction was performed for the complete genomic sequence with the RAST annotation server [39], followed by InterProScan [40] search and BLASTP search using nr database for validation. Genomic information, such as nucleotide variations and circular representations, was analyzed with gview software [41].

### Pairwise alignment of chromosomal sequences

Pairwise alignment was performed by BLASTN and TBLASTN homology searches [42] followed by visualization of the aligned images with the ACT [43] or EMBOSS dottup program [44].

### BLAST atlas

A BLAST atlas was generated by a BLASTP homology search [42] using the gview program [41]. The atlas displays BLASTP comparison results. The visualized area shows that the length of similar genes covers at least 80% between *M. massiliense* JCM 15300 and other *Mycobacterium* spp.

### SNP analysis

To construct simulated paired-end reads from the available genomic sequences of *M. abscessus* group strains, SimSeq software [45] was used with “SimSeq.jar” and “SamToFastq.jar” commands with the following default parameter modifications: number of pairs of reads, “—read_number 2000000”; mean library insert size, “—insert_size 150”; and paired-end reads length of 120 mer, “−1 120 −2 120”. These parameters indicated that 4 million hypothetical 120-mer reads were generated without mutations or indels from the genomic sequences used for SNP identification. To generate short-read mapping data of all *M. abscessus* group strains compared with the reference chromosomal sequence of *M. massiliense* JCM 15300, bwasw [46] and samtools [47] software was used with the default parameters. All SNPs were extracted by VarScan v2.3.4 [48] with the default parameters. All SNPs were concatenated to generate a pseudo sequence for phylogenetic analysis. The DNA maximum-likelihood program (RAxML v7.25) [49] was used for phylogenetic analysis with 1,000-fold bootstrapping. FigTree v. 1.2.3 software was used to display the generated tree.

### Phylogenetic analysis

Nucleotide and amino acid sequences were aligned with mafft v6.86 [50] followed by phylogenetic analysis using the neighbor-joining method or maximum-likelihood method with 1,000-fold bootstrapping in clustalW2 [51] or RAxML v7.25 software [49]. FigTree v. 1.2.3 software was used to display the generated tree.

### PCR amplification

The PCR mixture contained approximately 1 ng of template DNA, 1× PrimeSTAR GXL Buffer (Takara Biochem. Shiga, Japan), 200 µM of each dNTP, 200 nM of each primer, and a total of 2.5 unit of PrimeSTAR GXL DNA polymerase (Takara Biochem.). The primer sequences for PCR amplification are shown in S4 Table. PCR was performed in 25 µl volumes under the following conditions: at 98°C for 20 sec followed by 30 cycles at 98°C for 15 sec, 65°C for 15 sec and 68°C for 1 min (for below 1.5 kb amplicons) or 5 min (for over 1.5 kb amplicons). Amplified PCR products were electrophoresed in 1.0% (w/v) agarose gel at 100 V and detected by staining with GelRed (Biotium Inc. Hayward, CA).

### Bacterial culture

The *M. abscessus* and *M. massiliense* type strains were cultured at 37°C in Middlebrook 7H9 broth (Difco) supplemented with 10% OADC (BD) and 0.05% Tween 80 under aerobic or microaerobic (6% aerobic O_2_ tension) conditions with AnaeroPack (Mitsubishi Gas Chemical Company, Inc., Tokyo, Japan). Growth was monitored by removing aliquots at the indicated time points and measuring the OD at 530 nm.

### Statistical analysis

The statistical test between MmGI-1 positive *M. massiliense* and *M. abscessus* was calculated by Fisher's Exact Test. Data of bacterial culture are expressed as mean ± standard error (SE) from 7 MmGI-1 positive *M. massiliense*, 8 MmGI-1 negative *M. massiliense* and 12 *M. abscessus* isolates. Statistical analysis was performed using the student's t-test. The t-test was used to investigate whether the means of two groups are statistically different from each other. Differences were considered significant with a p-value of <0.05 and 0.01.

### Nucleotide sequence accession numbers

The complete genomic sequence of *M. massiliense* JCM 15300 has been deposited into the DNA Data Bank of Japan (DDBJ; accession number: AP014547).

## Supporting Information

S1 Figure **Comparative analysis between the complete genomic sequence of the** ***M. massiliense*** **JCM 15300 strain and draft genomic sequences of** ***M. massiliense*** **CCUG 48898.** The upper dot plot represents synteny between JCM 15300 and CCUG 48898, and the yellow vertical bars indicate gap regions in the draft genome of CCUG 48898. The bottom table shows gaps between contigs in CCUG 48898.
(TIF)

S2 Figure **Genomic comparison and BLAST atlas of 3 clusters in the** ***M. abscessus*** **group.** Comparative analysis of *M. massiliense* JCM 15300 and *M. abscessus* ATCC 19977 using a BLASTN homology search visualized by the ACT program (middle) and a BLAST atlas of *M. massiliense* JCM 15300 and *M. abscessus* ATCC 19977. In the comparative analysis, the red and blue bars between chromosomal DNA sequences represent nucleotide matches in the forward and reverse directions, respectively. BLASTN match scores less than 999 are not shown. In the BLAST atlas, the coding regions of JCM 15300 or ATCC 19977 were aligned against those of other *M. abscessus* group strains using BLASTP, and the results are displayed as colored bars (as in Fig. 1A). The three yellow boxes represent prophages on each chromosome. Specific features are represented by characters: †, unique region in the massiliense cluster; •, unique region in JCM 15300; §, unique region in the abscessus cluster; ¶, MmGI-1 (also see blue bars in Fig. 1A).
(TIF)

S3 Figure **Visualization for** ***M. abscessus*** **group pan-genomes and core genomes.** A. Curve for pan-genomes and core genomes of *M. abscessus* group. The box plots indicate the pan- or core genome size for each genome comparison. The median values were connected to represent the relationship between genome number and gene cluster number. B. Curve for the new gene cluster number observed with every increase in the number of *M. abscessus* group genomes.
(TIF)

S4 Figure **Phylogenetic tree of mycolic acid cyclopropane synthetase domain (CMAS, pfam02353) proteins in** ***Mycobacterium*** **using the maximum-likelihood method with 1,000-fold bootstrapping.** The scale indicates that a branch length of 0.3 is 30 times as long as one that would show a 1% difference between the amino acid sequences at the beginning and end of the branch. The number at each branch node represents the bootstrapping value. The proteins in red indicate proteins that are conserved only in the massiliense cluster.
(TIF)

S1 Table **Mutation sites in the complete genomic sequence of** ***M. massiliense*** **JCM 15300 compared with those in draft genomic sequences of** ***M. massiliense*** **CCUG 48898**.
(PDF)

S2 Table **The unique gene loci in** ***M. massiliense*** **JCM15300.**
(PDF)

S3 Table **The deleted genes of massiliense and bolletii clusters among** ***M. abscessus*** **group.**
(PDF)

S4 Table **Oligonucleotide primer sequences used in PCR assays and the judging method for presence of MmGI-1 and other** ***M. massiliense*** **unique regions.**
(PDF)

S5 Table **Isolates analyzed in the present study and results of conventional PCR based detection against MmGI-1 and other** ***M. massiliense*** **unique regions.**
(PDF)
