# Supplementary figures and images for: Complete Genome Sequence and Comparative Genomic Analysis of Mycobacterium massiliense JCM 15300 in the Mycobacterium abscessus Group Reveal a Conserved Genomic Island MmGI-1 Related to Putative Lipid Metabolism
Source: PLoS One. 2014 Dec 11;9(12):e114848. doi: 10.1371/journal.pone.0114848 (PMC4263727; doi:10.1371/journal.pone.0114848)

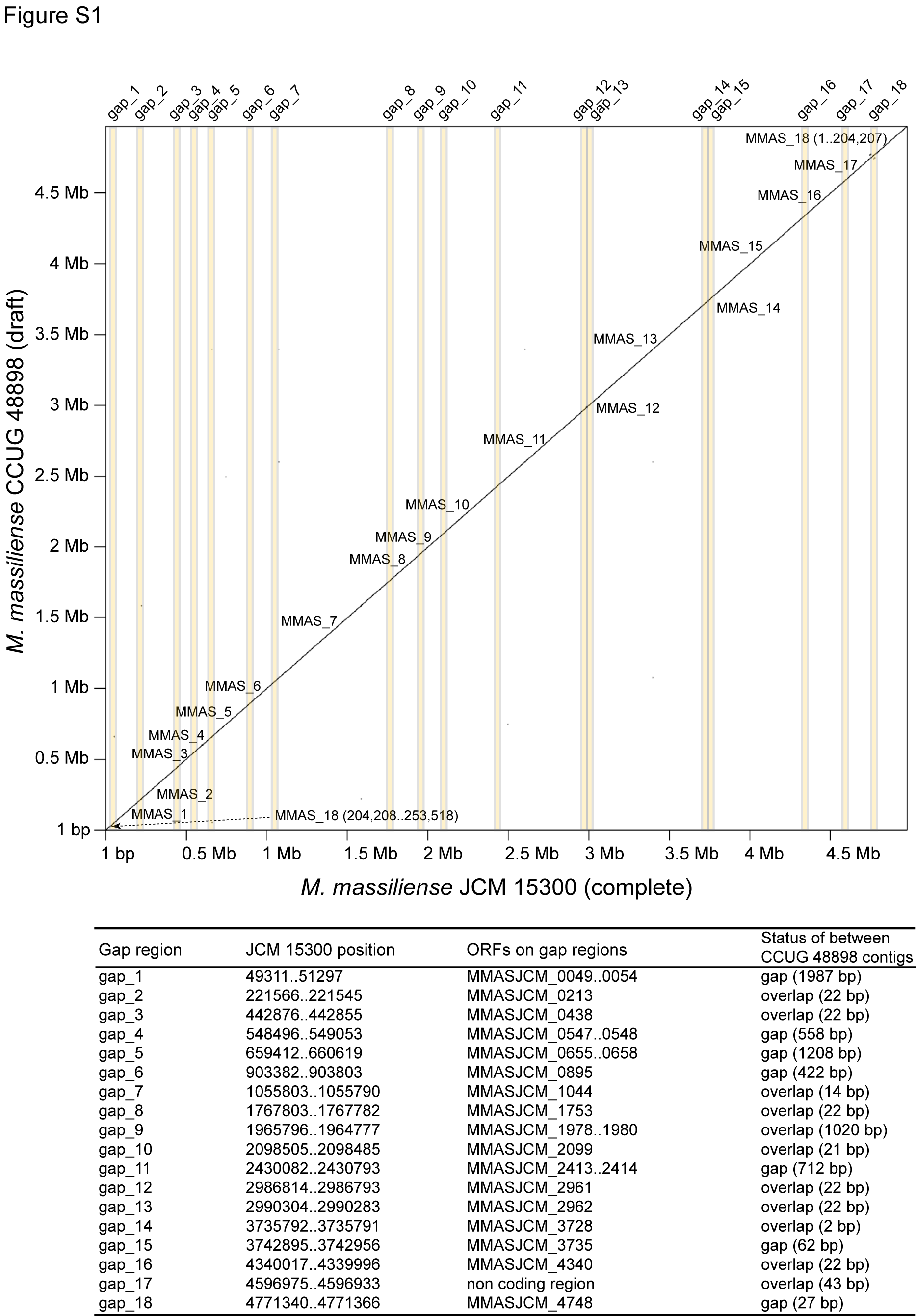

Supplement: S1 Figure — Comparative analysis between the complete genomic sequence of the M. massiliense JCM 15300 strain and draft genomic sequences of M. massiliense CCUG 48898. The upper dot plot represents synteny between JCM 15300 and CCUG 48898, and the yellow vertical bars indicate gap regions in the draft genome of CCUG 48898. The bottom table shows gaps between contigs in CCUG 48898. (TIF) [file pone.0114848.s001.tif]

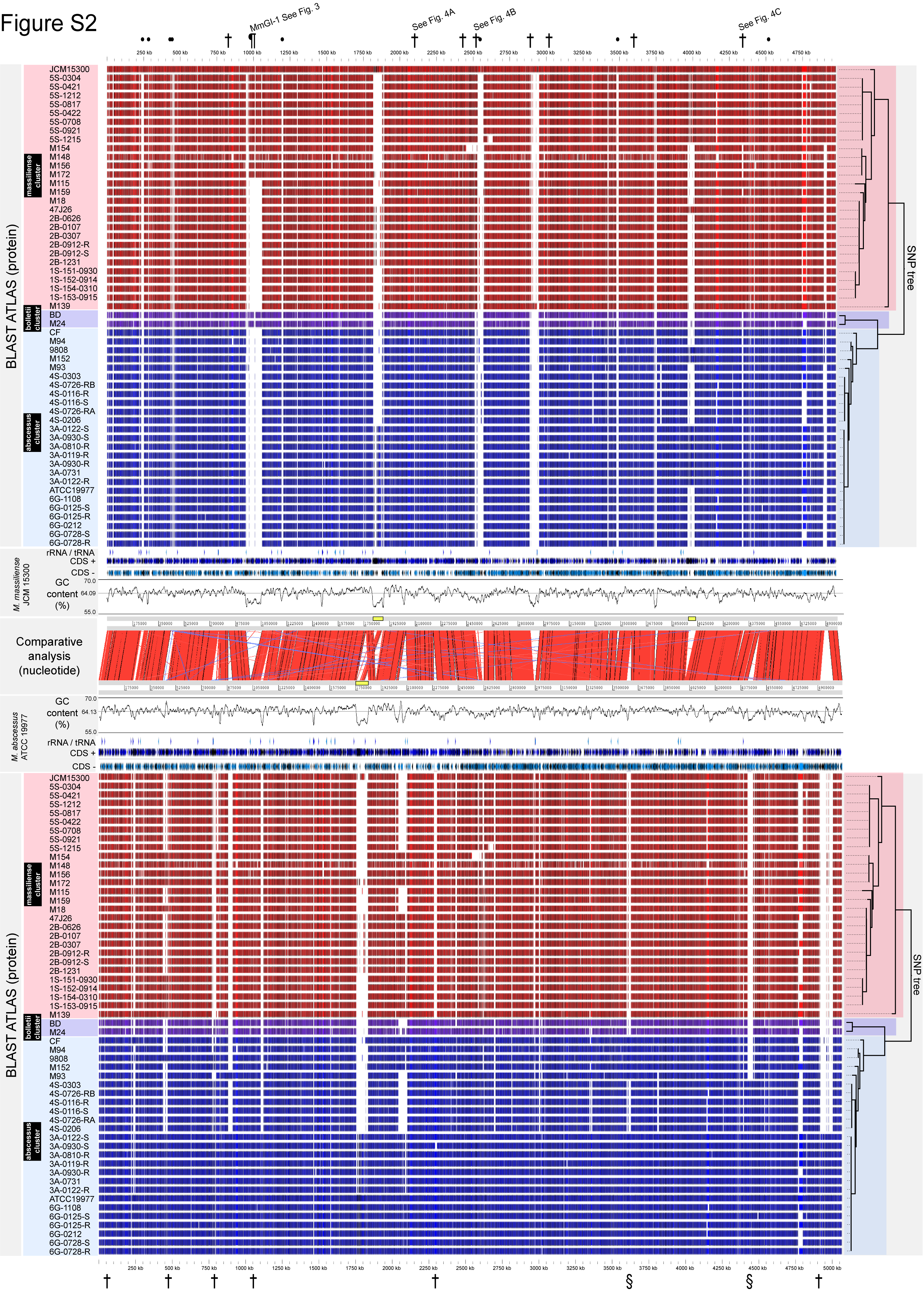

Supplement: S2 Figure — Genomic comparison and BLAST atlas of 3 clusters in the M. abscessus group. Comparative analysis of M. massiliense JCM 15300 and M. abscessus ATCC 19977 using a BLASTN homology search visualized by the ACT program (middle) and a BLAST atlas of M. massiliense JCM 15300 and M. abscessus ATCC 19977. In the comparative analysis, the red and blue bars between chromosomal DNA sequences represent nucleotide matches in the forward and reverse directions, respectively. BLASTN match scores less than 999 are not shown. In the BLAST atlas, the coding regions of JCM 15300 or ATCC 19977 were aligned against those of other M. abscessus group strains using BLASTP, and the results are displayed as colored bars (as in Fig. 1A). The three yellow boxes represent prophages on each chromosome. Specific features are represented by characters: †, unique region in the massiliense cluster; •, unique region in JCM 15300; §, unique region in the abscessus cluster; ¶, MmGI-1 (also see blue bars in Fig. 1A). (TIF) [file pone.0114848.s002.tif]

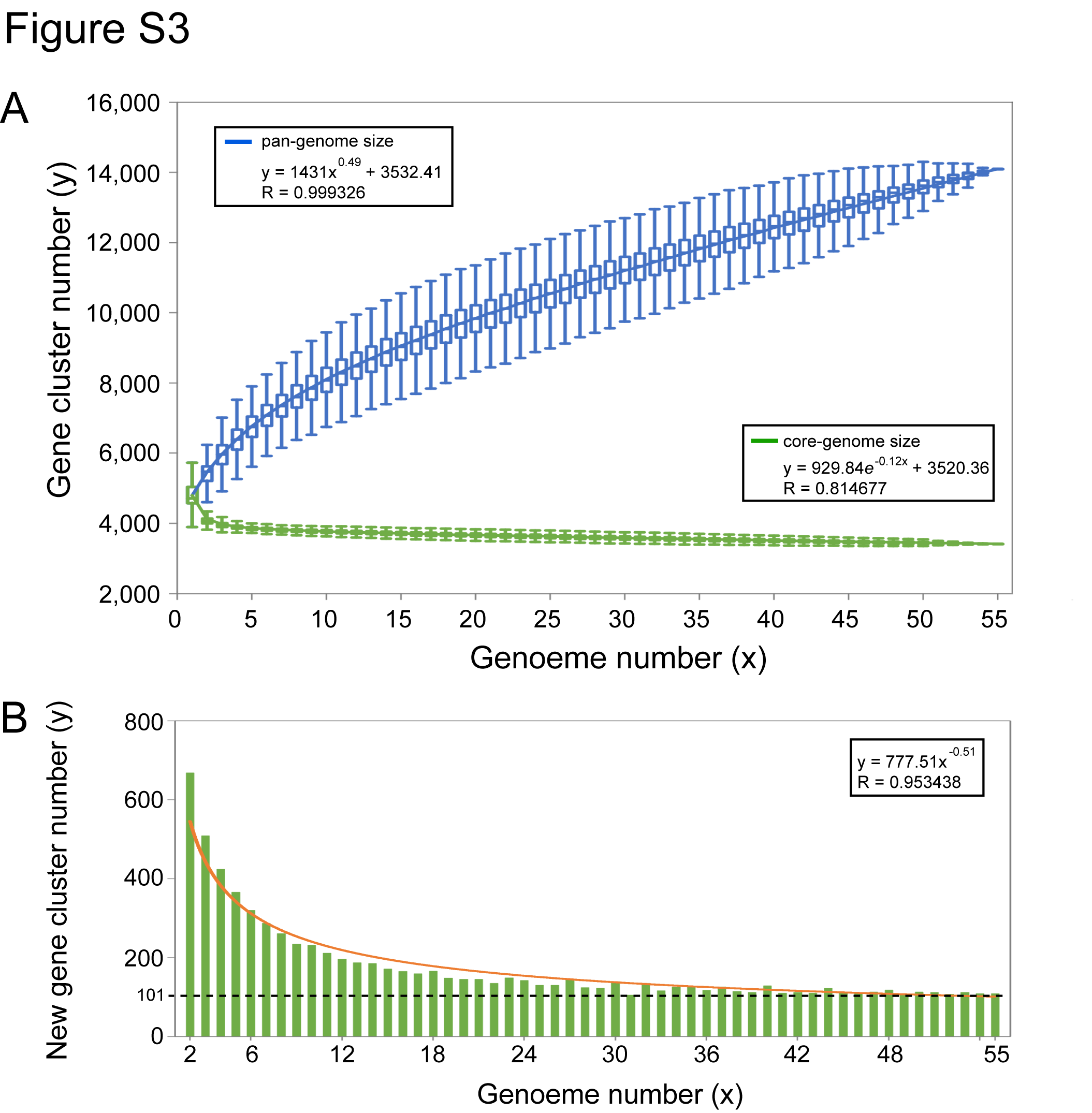

Supplement: S3 Figure — Visualization for M. abscessus group pan-genomes and core genomes. A. Curve for pan-genomes and core genomes of M. abscessus group. The box plots indicate the pan- or core genome size for each genome comparison. The median values were connected to represent the relationship between genome number and gene cluster number. B. Curve for the new gene cluster number observed with every increase in the number of M. abscessus group genomes. (TIF) [file pone.0114848.s003.tif]

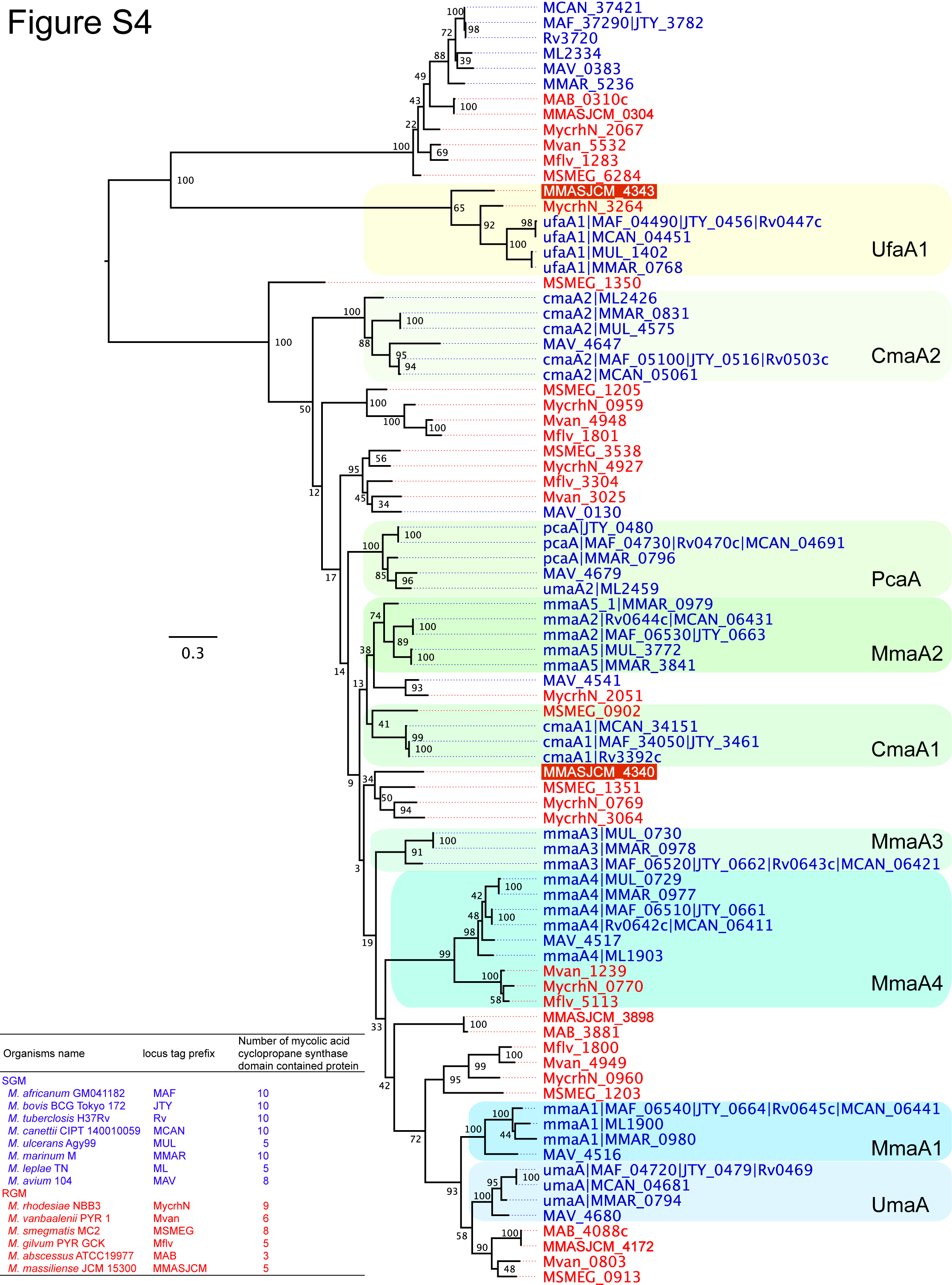

Supplement: S4 Figure — Phylogenetic tree of mycolic acid cyclopropane synthetase domain (CMAS, pfam02353) proteins in Mycobacterium using the maximum-likelihood method with 1,000-fold bootstrapping. The scale indicates that a branch length of 0.3 is 30 times as long as one that would show a 1% difference between the amino acid sequences at the beginning and end of the branch. The number at each branch node represents the bootstrapping value. The proteins in red indicate proteins that are conserved only in the massiliense cluster. (TIF) [file pone.0114848.s004.tif]
