# Supplementary material for: Complete Genome Sequence and Comparative Genomic Analysis of Mycobacterium massiliense JCM 15300 in the Mycobacterium abscessus Group Reveal a Conserved Genomic Island MmGI-1 Related to Putative Lipid Metabolism
Source: PLoS One. 2014 Dec 11;9(12):e114848. doi: 10.1371/journal.pone.0114848 (PMC4263727; doi:10.1371/journal.pone.0114848)
Supplement: S1 Table — Mutation sites in the complete genomic sequence of M. massiliense JCM 15300 compared with those in draft genomic sequences of M. massiliense CCUG 48898. (PDF) [file pone.0114848.s005.pdf]

Table S1. Mutation site between complete genome sequence of *M. massiliense* JCM 15300 and draft assembly of *M. massiliense* CCUG 48898.

JCM 15300 position	JCM 15300 nucleotide	CCUG 48898 nucleotide	Accession number of CCUG 48898 contig	CCUG 48898 position	Nucleotide mutation event of CCUG 48898	Amino acid mutation event of CCUG 48898	Location	strand	Synonym	Product
47437	T	C	NZ_AHAR01000018	251644	SNP (transition)	synonymous	47357..47665	+	MMASJCM_0047	Protein PE35, involved in regulation of esxAB expression
47467	G	C	NZ_AHAR01000018	251674	SNP (transversion)	synonymous	47357..47665	+	MMASJCM_0047	Protein PE35, involved in regulation of esxAB expression
47473	G	C	NZ_AHAR01000018	251680	SNP (transversion)	synonymous	47357..47665	+	MMASJCM_0047	Protein PE35, involved in regulation of esxAB expression
108736	–	GAGGCGCCC	NZ_AHAR01000001	57441..57449	Indel (+9)	insertion	108627..108794	+	MMASJCM_0109	hypothetical protein
112190..112222	CGGGCGCAGGCGTCTCGGCGGG CTTCGCCGCGG	–	NZ_AHAR01000001	60902	Indel (−33)	deletion	111810..112448	−	MMASJCM_0113	Alginate regulatory protein AlgP
220765	–	A	NZ_AHAR01000001	169446	Indel (+1)	frame shift (truncation)	219553..220890	+	MMASJCM_0212	hypothetical protein
221444	T	C	NZ_AHAR01000001	170125	SNP (transition)	synonymous	220962..222785	+	MMASJCM_0213	hypothetical protein
221447	C	G	NZ_AHAR01000001	170128	SNP (transversion)	synonymous	220962..222785	+	MMASJCM_0213	hypothetical protein
442269	C	T	NZ_AHAR01000002	220725	SNP (transition)	synonymous	441745..442500	+	MMASJCM_0437	Probable acetyl-CoA acetyltransferase FadA5
442290	G	A	NZ_AHAR01000002	220746	SNP (transition)	synonymous	441745..442500	+	MMASJCM_0437	Probable acetyl-CoA acetyltransferase FadA5
442326	G	A	NZ_AHAR01000002	220782	SNP (transition)	synonymous	441745..442500	+	MMASJCM_0437	Probable acetyl-CoA acetyltransferase FadA5
442372	A	G	NZ_AHAR01000002	220828	SNP (transition)	nonsynonymous (N -> D)	441745..442500	+	MMASJCM_0437	Probable acetyl-CoA acetyltransferase FadA5
442392	C	T	NZ_AHAR01000002	220848	SNP (transition)	synonymous	441745..442500	+	MMASJCM_0437	Probable acetyl-CoA acetyltransferase FadA5
442416	C	T	NZ_AHAR01000002	220872	SNP (transition)	synonymous	441745..442500	+	MMASJCM_0437	Probable acetyl-CoA acetyltransferase FadA5
442436	–	G	NZ_AHAR01000002	220893	Indel (+1)	frame shift (truncation)	441745..442500	+	MMASJCM_0437	Probable acetyl-CoA acetyltransferase FadA5
442649	T	C	NZ_AHAR01000002	221106	SNP (transition)	synonymous	442500..442904	+	MMASJCM_0438	Probable acetyl-CoA acetyltransferase FadA5
442688	T	C	NZ_AHAR01000002	221145	SNP (transition)	synonymous	442500..442904	+	MMASJCM_0438	Probable acetyl-CoA acetyltransferase FadA5
442700	A	G	NZ_AHAR01000002	221157	SNP (transition)	synonymous	442500..442904	+	MMASJCM_0438	Probable acetyl-CoA acetyltransferase FadA5
442706	T	C	NZ_AHAR01000002	221163	SNP (transition)	synonymous	442500..442904	+	MMASJCM_0438	Probable acetyl-CoA acetyltransferase FadA5
442707	G	T	NZ_AHAR01000002	221164	SNP (transversion)	synonymous	442500..442904	+	MMASJCM_0438	Probable acetyl-CoA acetyltransferase FadA5
599659	A	G	NZ_AHAR01000004	50167	SNP (transition)	synonymous	599134..600330	−	MMASJCM_0602	Probable acetyl-CoA acetyltransferase FadA5
599806	A	G	NZ_AHAR01000004	50314	SNP (transition)	synonymous	599134..600330	−	MMASJCM_0602	Probable acetyl-CoA acetyltransferase FadA5
599956	A	G	NZ_AHAR01000004	50464	SNP (transition)	synonymous	599134..600330	−	MMASJCM_0602	Probable acetyl-CoA acetyltransferase FadA5
599968	C	T	NZ_AHAR01000004	50476	SNP (transition)	synonymous	599134..600330	−	MMASJCM_0602	Probable acetyl-CoA acetyltransferase FadA5
600061	A	G	NZ_AHAR01000004	50569	SNP (transition)	synonymous	599134..600330	−	MMASJCM_0602	Probable acetyl-CoA acetyltransferase FadA5
600093	C	T	NZ_AHAR01000004	50601	SNP (transition)	nonsynonymous (A -> T)	599134..600330	−	MMASJCM_0602	Probable acetyl-CoA acetyltransferase FadA5
600172	A	G	NZ_AHAR01000004	50680	SNP (transition)	synonymous	599134..600330	−	MMASJCM_0602	Probable acetyl-CoA acetyltransferase FadA5
600202	C	G	NZ_AHAR01000004	50710	SNP (transversion)	synonymous	599134..600330	−	MMASJCM_0602	Probable acetyl-CoA acetyltransferase FadA5
600229	C	T	NZ_AHAR01000004	50737	SNP (transition)	synonymous	599134..600330	−	MMASJCM_0602	Probable acetyl-CoA acetyltransferase FadA5
600232	A	G	NZ_AHAR01000004	50740	SNP (transition)	synonymous	599134..600330	−	MMASJCM_0602	Probable acetyl-CoA acetyltransferase FadA5
600241	G	C	NZ_AHAR01000004	50749	SNP (transversion)	synonymous	599134..600330	−	MMASJCM_0602	Probable acetyl-CoA acetyltransferase FadA5
600304	C	T	NZ_AHAR01000004	50812	SNP (transition)	synonymous	599134..600330	−	MMASJCM_0602	Probable acetyl-CoA acetyltransferase FadA5
600955	–	C	NZ_AHAR01000004	51464	Indel (+1)	frame shift	600548..601807	+	MMASJCM_0603	Putative cytochrome P450 IgrA
600975	–	T	NZ_AHAR01000004	51485	Indel (+1)	frame shift (stop codon)	600548..601807	+	MMASJCM_0603	Putative cytochrome P450 IgrA
601067	–	C	NZ_AHAR01000004	51578	Indel (+1)	frame shift	600548..601807	+	MMASJCM_0603	Putative cytochrome P450 IgrA
601456	G	A	NZ_AHAR01000004	51967	SNP (transition)	synonymous	600548..601807	+	MMASJCM_0603	Putative cytochrome P450 IgrA
601501	C	T	NZ_AHAR01000004	52012	SNP (transition)	synonymous	600548..601807	+	MMASJCM_0603	Putative cytochrome P450 IgrA
601534	T	C	NZ_AHAR01000004	52045	SNP (transition)	synonymous	600548..601807	+	MMASJCM_0603	Putative cytochrome P450 IgrA
601543	G	A	NZ_AHAR01000004	52054	SNP (transition)	nonsynonymous (D -> E)	600548..601807	+	MMASJCM_0603	Putative cytochrome P450 IgrA
601546	T	G	NZ_AHAR01000004	52057	SNP (transversion)	synonymous	600548..601807	+	MMASJCM_0603	Putative cytochrome P450 IgrA
601549	G	C	NZ_AHAR01000004	52060	SNP (transversion)	synonymous	600548..601807	+	MMASJCM_0603	Putative cytochrome P450 IgrA
601564	T	C	NZ_AHAR01000004	52075	SNP (transition)	synonymous	600548..601807	+	MMASJCM_0603	Putative cytochrome P450 IgrA
601576	C	A	NZ_AHAR01000004	52087	SNP (transversion)	synonymous	600548..601807	+	MMASJCM_0603	Putative cytochrome P450 IgrA
601594	G	A	NZ_AHAR01000004	52105	SNP (transition)	synonymous	600548..601807	+	MMASJCM_0603	Putative cytochrome P450 IgrA
601618	T	C	NZ_AHAR01000004	52129	SNP (transition)	synonymous	600548..601807	+	MMASJCM_0603	Putative cytochrome P450 IgrA
601636	C	T	NZ_AHAR01000004	52147	SNP (transition)	synonymous	600548..601807	+	MMASJCM_0603	Putative cytochrome P450 IgrA
601651	G	C	NZ_AHAR01000004	52162	SNP (transversion)	synonymous	600548..601807	+	MMASJCM_0603	Putative cytochrome P450 IgrA
601652	T	A	NZ_AHAR01000004	52163	SNP (transversion)	synonymous	600548..601807	+	MMASJCM_0603	Putative cytochrome P450 IgrA
601663	C	T	NZ_AHAR01000004	52174	SNP (transition)	synonymous	600548..601807	+	MMASJCM_0603	Putative cytochrome P450 IgrA
856922	–	T	NZ_AHAR01000005	196318	Indel (+1)	frame shift (truncation)	852073..863172	+	MMASJCM_0856	Malonyl CoA-acyl carrier protein transacylase
903048	T	C	NZ_AHAR01000005	242444	SNP (transition)	synonymous	902977..904929	+	MMASJCM_0895	hypothetical protein
903845..903847	CGA	–	NZ_AHAR01000006	42	Indel (−3)	deletion	902977..904929	+	MMASJCM_0895	hypothetical protein
904877	–	CGGCTCCTCGTCCAG	NZ_AHAR01000006	1073..1087	Indel (+15)	insertion	902977..904929	+	MMASJCM_0895	hypothetical protein
1111655	–	A	NZ_AHAR01000007	55867	Indel (+1)	frame shift (truncation)	1111195..1112730	+	MMASJCM_1102	sensor histidine kinase

1399166	-	GGTGCCGCTGGGGCCGGTGCCG GTGCG	NZ_AHAR01000007	342268..342294	Indel (+27)	insertion	1395656..1399342	-	MMASJCM_1413	Dihydrolipoamide succinyltransferase component (E2) of 2-oxoglutarate dehydrogenase complex 2-oxoglutarate dehydrogenase E1 component
1586566	T	C	NZ_AHAR01000007	529720	SNP (transition)	variable mutation	1585184..1588654	+	MMASJCM_1576	hypothetical protein
1586568	T	C	NZ_AHAR01000007	529722	SNP (transition)	variable mutation	1585184..1588654	+	MMASJCM_1576	hypothetical protein
1586569	G	A	NZ_AHAR01000007	529723	SNP (transition)	variable mutation	1585184..1588654	+	MMASJCM_1576	hypothetical protein
1586575	-	C	NZ_AHAR01000007	529730	Indel (+1)	variable mutation	1585184..1588654	+	MMASJCM_1576	hypothetical protein
1586577	A	T	NZ_AHAR01000007	529732	SNP (transversion)	variable mutation	1585184..1588654	+	MMASJCM_1576	hypothetical protein
1586578	-	T	NZ_AHAR01000007	529734	Indel (+1)	variable mutation	1585184..1588654	+	MMASJCM_1576	hypothetical protein
1586580	-	AACA	NZ_AHAR01000007	529737..529740	Indel (+4)	variable mutation	1585184..1588654	+	MMASJCM_1576	hypothetical protein
1586584	T	G	NZ_AHAR01000007	529744	SNP (transversion)	variable mutation	1585184..1588654	+	MMASJCM_1576	hypothetical protein
1586589	-	G	NZ_AHAR01000007	529750	Indel (+1)	variable mutation	1585184..1588654	+	MMASJCM_1576	hypothetical protein
1586591	-	AT	NZ_AHAR01000007	529753..529754	Indel (+2)	variable mutation	1585184..1588654	+	MMASJCM_1576	hypothetical protein
1586596..1586597	GG	-	NZ_AHAR01000007	529758	Indel (-2)	variable mutation	1585184..1588654	+	MMASJCM_1576	hypothetical protein
1586599	G	C	NZ_AHAR01000007	529760	SNP (transversion)	variable mutation	1585184..1588654	+	MMASJCM_1576	hypothetical protein
1586602	T	-	NZ_AHAR01000007	529762	Indel (-1)	variable mutation	1585184..1588654	+	MMASJCM_1576	hypothetical protein
1586606	G	C	NZ_AHAR01000007	529766	SNP (transversion)	variable mutation	1585184..1588654	+	MMASJCM_1576	hypothetical protein
1586611	G	A	NZ_AHAR01000007	529771	SNP (transition)	variable mutation	1585184..1588654	+	MMASJCM_1576	hypothetical protein
1586613	-	AGAT	NZ_AHAR01000007	529774..529777	Indel (+4)	variable mutation	1585184..1588654	+	MMASJCM_1576	hypothetical protein
1586616	C	T	NZ_AHAR01000007	529780	SNP (transition)	variable mutation	1585184..1588654	+	MMASJCM_1576	hypothetical protein
1586617	C	G	NZ_AHAR01000007	529781	SNP (transversion)	variable mutation	1585184..1588654	+	MMASJCM_1576	hypothetical protein
1586618	-	GC	NZ_AHAR01000007	529783..529784	Indel (+2)	variable mutation	1585184..1588654	+	MMASJCM_1576	hypothetical protein
1586620	T	G	NZ_AHAR01000007	529786	SNP (transversion)	variable mutation	1585184..1588654	+	MMASJCM_1576	hypothetical protein
1586623	-	CC	NZ_AHAR01000007	529790..529791	Indel (+2)	variable mutation	1585184..1588654	+	MMASJCM_1576	hypothetical protein
1586627	-	CAA	NZ_AHAR01000007	529796..529798	Indel (+3)	variable mutation	1585184..1588654	+	MMASJCM_1576	hypothetical protein
1586628	-	C	NZ_AHAR01000007	529800	Indel (+1)	variable mutation	1585184..1588654	+	MMASJCM_1576	hypothetical protein
1586630	-	CGT	NZ_AHAR01000007	529803..529807	Indel (+3)	variable mutation	1585184..1588654	+	MMASJCM_1576	hypothetical protein
1586632	-	T	NZ_AHAR01000007	529807	Indel (+1)	variable mutation	1585184..1588654	+	MMASJCM_1576	hypothetical protein
1586635	-	TCC	NZ_AHAR01000007	529811..529813	Indel (+3)	variable mutation	1585184..1588654	+	MMASJCM_1576	hypothetical protein
1586641	C	-	NZ_AHAR01000007	529818	Indel (-1)	variable mutation	1585184..1588654	+	MMASJCM_1576	hypothetical protein
1586643	-	A	NZ_AHAR01000007	529821	Indel (+1)	variable mutation	1585184..1588654	+	MMASJCM_1576	hypothetical protein
1586644	C	G	NZ_AHAR01000007	529822	SNP (transversion)	variable mutation	1585184..1588654	+	MMASJCM_1576	hypothetical protein
1586646	-	G	NZ_AHAR01000007	529825	Indel (+1)	variable mutation	1585184..1588654	+	MMASJCM_1576	hypothetical protein
1586648	C	A	NZ_AHAR01000007	529827	SNP (transversion)	variable mutation	1585184..1588654	+	MMASJCM_1576	hypothetical protein
1586650	C	-	NZ_AHAR01000007	529828	Indel (-1)	variable mutation	1585184..1588654	+	MMASJCM_1576	hypothetical protein
1586659	C	T	NZ_AHAR01000007	529837	SNP (transition)	variable mutation	1585184..1588654	+	MMASJCM_1576	hypothetical protein
1586662	A	G	NZ_AHAR01000007	529840	SNP (transition)	variable mutation	1585184..1588654	+	MMASJCM_1576	hypothetical protein
1586665	C	G	NZ_AHAR01000007	529843	SNP (transversion)	variable mutation	1585184..1588654	+	MMASJCM_1576	hypothetical protein
1586668	C	T	NZ_AHAR01000007	529846	SNP (transition)	variable mutation	1585184..1588654	+	MMASJCM_1576	hypothetical protein
1586677	A	G	NZ_AHAR01000007	529855	SNP (transition)	variable mutation	1585184..1588654	+	MMASJCM_1576	hypothetical protein
1586681..1586683	CAG	-	NZ_AHAR01000007	529858	Indel (-3)	variable mutation	1585184..1588654	+	MMASJCM_1576	hypothetical protein
1586710	C	T	NZ_AHAR01000007	529885	SNP (transition)	variable mutation	1585184..1588654	+	MMASJCM_1576	hypothetical protein
1586730	C	G	NZ_AHAR01000007	529905	SNP (transversion)	variable mutation	1585184..1588654	+	MMASJCM_1576	hypothetical protein
1586731	T	C	NZ_AHAR01000007	529906	SNP (transition)	variable mutation	1585184..1588654	+	MMASJCM_1576	hypothetical protein
1586845	C	T	NZ_AHAR01000007	530020	SNP (transition)	variable mutation	1585184..1588654	+	MMASJCM_1576	hypothetical protein
1586847	A	-	NZ_AHAR01000007	530021	Indel (-1)	variable mutation	1585184..1588654	+	MMASJCM_1576	hypothetical protein
1586849	T	G	NZ_AHAR01000007	530023	SNP (transversion)	variable mutation	1585184..1588654	+	MMASJCM_1576	hypothetical protein
1586851	A	C	NZ_AHAR01000007	530025	SNP (transversion)	variable mutation	1585184..1588654	+	MMASJCM_1576	hypothetical protein
1586853	A	G	NZ_AHAR01000007	530027	SNP (transition)	variable mutation	1585184..1588654	+	MMASJCM_1576	hypothetical protein
1586854	-	T	NZ_AHAR01000007	530029	Indel (+1)	variable mutation	1585184..1588654	+	MMASJCM_1576	hypothetical protein
1586855	A	G	NZ_AHAR01000007	530030	SNP (transition)	variable mutation	1585184..1588654	+	MMASJCM_1576	hypothetical protein
1586856	A	G	NZ_AHAR01000007	530031	SNP (transition)	variable mutation	1585184..1588654	+	MMASJCM_1576	hypothetical protein
1586863	C	T	NZ_AHAR01000007	530038	SNP (transition)	variable mutation	1585184..1588654	+	MMASJCM_1576	hypothetical protein
1586867	T	A	NZ_AHAR01000007	530042	SNP (transversion)	variable mutation	1585184..1588654	+	MMASJCM_1576	hypothetical protein
1586870	G	A	NZ_AHAR01000007	530045	SNP (transition)	variable mutation	1585184..1588654	+	MMASJCM_1576	hypothetical protein
1587136	T	C	NZ_AHAR01000007	530311	SNP (transition)	variable mutation	1585184..1588654	+	MMASJCM_1576	hypothetical protein
1587220	C	T	NZ_AHAR01000007	530395	SNP (transition)	variable mutation	1585184..1588654	+	MMASJCM_1576	hypothetical protein
1587223	G	C	NZ_AHAR01000007	530398	SNP (transversion)	variable mutation	1585184..1588654	+	MMASJCM_1576	hypothetical protein
1587343	C	T	NZ_AHAR01000007	530518	SNP (transition)	variable mutation	1585184..1588654	+	MMASJCM_1576	hypothetical protein

1587364	T	C	NZ_AHAR01000007	530539	SNP (transition)	variable mutation	1585184..1588654	+	MMASJCM_1576	hypothetical protein
1587385	T	G	NZ_AHAR01000007	530560	SNP (transversion)	variable mutation	1585184..1588654	+	MMASJCM_1576	hypothetical protein
1587388	C	G	NZ_AHAR01000007	530563	SNP (transversion)	variable mutation	1585184..1588654	+	MMASJCM_1576	hypothetical protein
1587399	C	T	NZ_AHAR01000007	530574	SNP (transition)	variable mutation	1585184..1588654	+	MMASJCM_1576	hypothetical protein
1587400	A	C	NZ_AHAR01000007	530575	SNP (transversion)	variable mutation	1585184..1588654	+	MMASJCM_1576	hypothetical protein
1587406	C	T	NZ_AHAR01000007	530581	SNP (transition)	variable mutation	1585184..1588654	+	MMASJCM_1576	hypothetical protein
1587409	C	G	NZ_AHAR01000007	530584	SNP (transversion)	variable mutation	1585184..1588654	+	MMASJCM_1576	hypothetical protein
1587428	G	A	NZ_AHAR01000007	530603	SNP (transition)	variable mutation	1585184..1588654	+	MMASJCM_1576	hypothetical protein
1587436	–	GGTGTGG	NZ_AHAR01000007	530612..530618	Indel (+7)	variable mutation	1585184..1588654	+	MMASJCM_1576	hypothetical protein
1587439	–	AT	NZ_AHAR01000007	530622..530623	Indel (+2)	variable mutation	1585184..1588654	+	MMASJCM_1576	hypothetical protein
1946214	C	–	NZ_AHAR01000008	178432	Indel (–1)	non coding region	non coding region			
1946386	A	G	NZ_AHAR01000008	178604	SNP (transition)	non coding region	non coding region			
1946389	C	A	NZ_AHAR01000008	178607	SNP (transversion)	non coding region	non coding region			
1946409..1946429	CAGCCGGCGCGGGAGCCGGGG	–	NZ_AHAR01000008	178626	Indel (–21)	non coding region	non coding region			
1946438..1946449	GCGGCTGGTGCG	–	NZ_AHAR01000008	178634	Indel (–12)	non coding region	non coding region			
1957223..1957240	CCGCGGCGTCCGGGACCT	–	NZ_AHAR01000008	189419	Indel (–18)	deletion	1956558..1957637	+	MMASJCM_1970	Glycerate kinase
1965870	C	G	NZ_AHAR01000009	1094	SNP (transversion)	nonsynonymous (K -> N)	1965702..1967342	–	MMASJCM_1980	Ubiquinol--cytochrome c reductase, cytochrome B subunit
1965911	C	G	NZ_AHAR01000009	1135	SNP (transversion)	nonsynonymous (A -> P)	1965702..1967342	–	MMASJCM_1980	Ubiquinol--cytochrome c reductase, cytochrome B subunit
2193158	A	T	NZ_AHAR01000010	94109	SNP (transversion)	synonymous	2191949..2193520	–	MMASJCM_2185	PPE family protein
2193523	G	C	NZ_AHAR01000010	94474	SNP (transversion)	non coding region	non coding region			
2193553	G	A	NZ_AHAR01000010	94504	SNP (transition)	synonymous	2193526..2193834	–	MMASJCM_2186	Protein PE35, involved in regulation of esxAB expression
2193718	G	C	NZ_AHAR01000010	94669	SNP (transversion)	synonymous	2193526..2193834	–	MMASJCM_2186	Protein PE35, involved in regulation of esxAB expression
2193724	G	C	NZ_AHAR01000010	94675	SNP (transversion)	synonymous	2193526..2193834	–	MMASJCM_2186	Protein PE35, involved in regulation of esxAB expression
2193754	G	A	NZ_AHAR01000010	94705	SNP (transition)	synonymous	2193526..2193834	–	MMASJCM_2186	Protein PE35, involved in regulation of esxAB expression
2222544	–	G	NZ_AHAR01000010	123496	Indel (+1)	frame shift (truncation)	2222216..2222560	+	MMASJCM_2207	Uncharacterized protein conserved in bacteria
2496668	A	G	NZ_AHAR01000011	65876	SNP (transition)	synonymous	2495990..2496973	–	MMASJCM_2489	Probable acyl-ACP desaturase, Stearoyl-ACP desaturase
2602787	T	C	NZ_AHAR01000011	171995	SNP (transition)	synonymous	2602424..2604118	–	MMASJCM_2594	Cytochrome c oxidase polypeptide I
2602790	C	G	NZ_AHAR01000011	171998	SNP (transversion)	nonsynonymous (S -> A)	2602424..2604118	–	MMASJCM_2594	Cytochrome c oxidase polypeptide I
2602792	A	C	NZ_AHAR01000011	172000	SNP (transversion)	synonymous	2602424..2604118	–	MMASJCM_2594	Cytochrome c oxidase polypeptide I
2602796	G	A	NZ_AHAR01000011	172004	SNP (transition)	synonymous	2602424..2604118	–	MMASJCM_2594	Cytochrome c oxidase polypeptide I
2603065	A	G	NZ_AHAR01000011	172273	SNP (transition)	synonymous	2602424..2604118	–	MMASJCM_2594	Cytochrome c oxidase polypeptide I
2603069	C	A	NZ_AHAR01000011	172277	SNP (transversion)	synonymous	2602424..2604118	–	MMASJCM_2594	Cytochrome c oxidase polypeptide I
2603111	C	G	NZ_AHAR01000011	172319	SNP (transversion)	synonymous	2602424..2604118	–	MMASJCM_2594	Cytochrome c oxidase polypeptide I
2603114	C	G	NZ_AHAR01000011	172322	SNP (transversion)	synonymous	2602424..2604118	–	MMASJCM_2594	Cytochrome c oxidase polypeptide I
2603117	C	A	NZ_AHAR01000011	172325	SNP (transversion)	synonymous	2602424..2604118	–	MMASJCM_2594	Cytochrome c oxidase polypeptide I
2603126	G	C	NZ_AHAR01000011	172334	SNP (transversion)	synonymous	2602424..2604118	–	MMASJCM_2594	Cytochrome c oxidase polypeptide I
2603129	A	G	NZ_AHAR01000011	172337	SNP (transition)	synonymous	2602424..2604118	–	MMASJCM_2594	Cytochrome c oxidase polypeptide I
2603130	T	A	NZ_AHAR01000011	172338	SNP (transversion)	synonymous	2602424..2604118	–	MMASJCM_2594	Cytochrome c oxidase polypeptide I
2603132	G	C	NZ_AHAR01000011	172340	SNP (transversion)	nonsynonymous (Y -> F)	2602424..2604118	–	MMASJCM_2594	Cytochrome c oxidase polypeptide I
2603171	G	C	NZ_AHAR01000011	172379	SNP (transversion)	synonymous	2602424..2604118	–	MMASJCM_2594	Cytochrome c oxidase polypeptide I
2603175	A	T	NZ_AHAR01000011	172383	SNP (transversion)	nonsynonymous (F -> Y)	2602424..2604118	–	MMASJCM_2594	Cytochrome c oxidase polypeptide I
2709579..2709580	TA	–	NZ_AHAR01000011	278786	Indel (–2)	non coding region	non coding region			
2990634..2990653	ACCCGCTTCGACACCGCCAT	–	NZ_AHAR01000011	351	Indel (–20)	frame shift (truncation)	2987313..2990807	+	MMASJCM_2962	Long-chain-fatty-acid--CoA ligase
3128864	–	CAAACCCGGATGAGCGCCAGCGAAGACCAT	NZ_AHAR01000013	138617..138646	Indel (+30)	insertion	3128814..3129986	+	MMASJCM_3124	acyl-CoA dehydrogenase domain protein
3137366	G	–	NZ_AHAR01000013	147147	Indel (–1)	frame shift (truncation)	3136691..3137542	+	MMASJCM_3133	hypothetical protein
3191312	–	A	NZ_AHAR01000013	201094	Indel (+1)	frame shift (truncation)	3190918..3191874	+	MMASJCM_3184	Permease of the drug metabolite transporter (DMT) superfamily
3192040	–	CCA	NZ_AHAR01000013	201823..201825	Indel (+3)	insertion	3191988..3192110	–	MMASJCM_3185	hypothetical protein
3217221	G	A	NZ_AHAR01000013	227015	SNP (transition)	synonymous	3216840..3217367	–	MMASJCM_3216	Carbonic anhydrase
3224069	–	ACGACC	NZ_AHAR01000013	233286..233291	Indel (+6)	insertion	3223932..3224348	+	MMASJCM_3226	hypothetical protein
3361131	A	G	NZ_AHAR01000013	370353	SNP (transition)	nonsynonymous (I -> V)	3361017..3361433	+	MMASJCM_3353	Probable acyl-ACP desaturase, Stearoyl-ACP desaturase
3361136	C	G	NZ_AHAR01000013	370358	SNP (transversion)	synonymous	3361017..3361433	+	MMASJCM_3353	Probable acyl-ACP desaturase, Stearoyl-ACP desaturase
3361195	G	C	NZ_AHAR01000013	370417	SNP (transversion)	nonsynonymous (C -> S)	3361017..3361433	+	MMASJCM_3353	Probable acyl-ACP desaturase, Stearoyl-ACP desaturase
3361202	G	C	NZ_AHAR01000013	370424	SNP (transversion)	synonymous	3361017..3361433	+	MMASJCM_3353	Probable acyl-ACP desaturase, Stearoyl-ACP desaturase
3361214	G	C	NZ_AHAR01000013	370436	SNP (transversion)	synonymous	3361017..3361433	+	MMASJCM_3353	Probable acyl-ACP desaturase, Stearoyl-ACP desaturase
3361216	G	A	NZ_AHAR01000013	370438	SNP (transition)	nonsynonymous (R -> K)	3361017..3361433	+	MMASJCM_3353	Probable acyl-ACP desaturase, Stearoyl-ACP desaturase
3361218	G	A	NZ_AHAR01000013	370440	SNP (transition)	nonsynonymous (V -> I)	3361017..3361433	+	MMASJCM_3353	Probable acyl-ACP desaturase, Stearoyl-ACP desaturase
3361220	A	C	NZ_AHAR01000013	370442	SNP (transversion)	nonsynonymous (V -> I)	3361017..3361433	+	MMASJCM_3353	Probable acyl-ACP desaturase, Stearoyl-ACP desaturase

3361223	C	G	NZ_AHAR01000013	370445	SNP (transversion)	synonymous	3361017..3361433	+	MMASJCM_3353	Probable acyl-ACP desaturase, Stearoyl-ACP desaturase
3361227	G	A	NZ_AHAR01000013	370449	SNP (transition)	nonsynonymous (V -> I)	3361017..3361433	+	MMASJCM_3353	Probable acyl-ACP desaturase, Stearoyl-ACP desaturase
3361253	C	T	NZ_AHAR01000013	370475	SNP (transition)	synonymous	3361017..3361433	+	MMASJCM_3353	Probable acyl-ACP desaturase, Stearoyl-ACP desaturase
3361260	G	T	NZ_AHAR01000013	370482	SNP (transversion)	nonsynonymous (A -> S)	3361017..3361433	+	MMASJCM_3353	Probable acyl-ACP desaturase, Stearoyl-ACP desaturase
3361262	C	G	NZ_AHAR01000013	370484	SNP (transversion)	nonsynonymous (A -> S)	3361017..3361433	+	MMASJCM_3353	Probable acyl-ACP desaturase, Stearoyl-ACP desaturase
3361337	A	G	NZ_AHAR01000013	370559	SNP (transition)	synonymous	3361017..3361433	+	MMASJCM_3353	Probable acyl-ACP desaturase, Stearoyl-ACP desaturase
3361340	A	G	NZ_AHAR01000013	370562	SNP (transition)	synonymous	3361017..3361433	+	MMASJCM_3353	Probable acyl-ACP desaturase, Stearoyl-ACP desaturase
3361350	A	G	NZ_AHAR01000013	370572	SNP (transition)	nonsynonymous (S -> G)	3361017..3361433	+	MMASJCM_3353	Probable acyl-ACP desaturase, Stearoyl-ACP desaturase
3361412	G	A	NZ_AHAR01000013	370634	SNP (transition)	synonymous	3361017..3361433	+	MMASJCM_3353	Probable acyl-ACP desaturase, Stearoyl-ACP desaturase
3361415..3361418	AG	-	NZ_AHAR01000013	370636	Indel (-2)	frame shift (truncation)	3361017..3361433	+	MMASJCM_3353	Probable acyl-ACP desaturase, Stearoyl-ACP desaturase
3361418	G	-	NZ_AHAR01000013	370638	Indel (-1)	frame shift (truncation)	3361017..3361433	+	MMASJCM_3353	Probable acyl-ACP desaturase, Stearoyl-ACP desaturase
3594115	C	G	NZ_AHAR01000013	603335	SNP (transversion)	nonsynonymous (E -> Q)	3593912..3594541	-	MMASJCM_3589	transcriptional regulator, IclR family
3594117	C	A	NZ_AHAR01000013	603337	SNP (transversion)	nonsynonymous (G -> L)	3593912..3594541	-	MMASJCM_3589	transcriptional regulator, IclR family
3594118	C	A	NZ_AHAR01000013	603338	SNP (transversion)	nonsynonymous (G -> L)	3593912..3594541	-	MMASJCM_3589	transcriptional regulator, IclR family
3594119	G	A	NZ_AHAR01000013	603339	SNP (transition)	nonsynonymous (D -> F)	3593912..3594541	-	MMASJCM_3589	transcriptional regulator, IclR family
3594120	T	A	NZ_AHAR01000013	603340	SNP (transversion)	nonsynonymous (D -> F)	3593912..3594541	-	MMASJCM_3589	transcriptional regulator, IclR family
3594121	C	A	NZ_AHAR01000013	603341	SNP (transversion)	nonsynonymous (D -> F)	3593912..3594541	-	MMASJCM_3589	transcriptional regulator, IclR family
4177363	-	G	NZ_AHAR01000015	434409	Indel (+1)	frame shift (truncation)	4167500..4177864	-	MMASJCM_4185	Long-chain-fatty-acid--CoA ligase
4382827	-	TAACGCTGGGTTGGCGGGCAATGCTGGGTTGGCGGGTAACGCTGGGTTGGCGGGCAATGCTGGGTTGGCGGGCAATGCTGGGTTGGCGGG	NZ_AHAR01000016	43024..43113	Indel (+90)	insertion	4382181..4383764	-	MMASJCM_4387	Proline sodium symporter PutP
4751778	-	CAA	NZ_AHAR01000017	154856..154858	Indel (+3)	insertion	4743910..4752057	-	MMASJCM_4747	Long-chain-fatty-acid--CoA ligase
