# Supplementary material for: Complete Genome Sequence and Comparative Genomic Analysis of Mycobacterium massiliense JCM 15300 in the Mycobacterium abscessus Group Reveal a Conserved Genomic Island MmGI-1 Related to Putative Lipid Metabolism
Source: PLoS One. 2014 Dec 11;9(12):e114848. doi: 10.1371/journal.pone.0114848 (PMC4263727; doi:10.1371/journal.pone.0114848)
Supplement: S2 Table — The unique gene loci in M. massiliense JCM15300. (PDF) [file pone.0114848.s006.pdf]

Table S2. The unique gene loci in *M. massiliense* JCM15300.

Gene_ID	Location at JCM 15300	Strand	Length	Product
MMASJCM_0221	232554..233921	-	455	integrase
MMASJCM_0225	237648..238013	+	121	transcriptional regulator
MMASJCM_0226	238206..239453	+	415	hypothetical protein
MMASJCM_0228	241293..241556	-	87	hypothetical protein
MMASJCM_0229	241653..242360	+	235	hypothetical protein
MMASJCM_0230	242468..243046	+	192	hypothetical protein
MMASJCM_0231	243070..243870	+	266	hypothetical protein
MMASJCM_0232	243938..244057	-	39	hypothetical protein
MMASJCM_0235	245472..245741	-	89	hypothetical protein
MMASJCM_0236	246143..248608	-	821	hypothetical protein
MMASJCM_0237	248605..248922	-	105	hypothetical protein
MMASJCM_0240	250602..250739	-	45	hypothetical protein
MMASJCM_0242	251336..251452	-	38	hypothetical protein
MMASJCM_0243	251449..251595	-	48	hypothetical protein
MMASJCM_0244	251624..251860	+	78	hypothetical protein
MMASJCM_0280	282806..282925	-	39	hypothetical protein
MMASJCM_0281	283333..283512	+	59	hypothetical protein
MMASJCM_0282	283532..285691	-	719	hypothetical protein
MMASJCM_0428	433050..433247	+	65	hypothetical protein
MMASJCM_0429	433435..434598	-	387	putative DNA-binding protein
MMASJCM_0435	439516..441177	+	553	sulfate permease
MMASJCM_0436	441331..441705	+	124	hypothetical protein
MMASJCM_0448	453064..454026	-	320	hypothetical protein
MMASJCM_0450	454684..454941	-	85	hypothetical protein
MMASJCM_0456	458244..458657	-	137	hypothetical protein
MMASJCM_0457	458836..460377	+	513	sulfate permease
MMASJCM_0458	460454..460867	+	137	putative transcriptional regulator
MMASJCM_1184	1191259..1191480	-	73	hypothetical protein
MMASJCM_1185	1192379..1192561	-	60	hypothetical protein
MMASJCM_1186	1192703..1192933	+	76	hypothetical protein
MMASJCM_1187	1192930..1193697	+	255	hypothetical protein
MMASJCM_1189	1195082..1195228	-	48	hypothetical protein
MMASJCM_1190	1195228..1195776	-	182	resolvase
MMASJCM_1191	1195989..1197131	-	380	hypothetical protein
MMASJCM_1192	1197340..1198923	-	527	putative ATP-binding protein
MMASJCM_1193	1199055..1199699	-	214	hypothetical protein
MMASJCM_1196	1202290..1202739	-	149	hypothetical protein
MMASJCM_2531	2531152..2531979	-	275	transcriptional regulator
MMASJCM_2532	2532176..2532469	+	97	hypothetical protein
MMASJCM_2533	2532466..2533305	+	279	short-chain dehydrogenase reductase SDR precursor
MMASJCM_2535	2534393..2535688	-	431	hypothetical protein
MMASJCM_2536	2535691..2536467	-	258	hypothetical protein
MMASJCM_2553	2560966..2561190	-	74	hypothetical protein
MMASJCM_2554	2561266..2562528	+	420	mobile element protein
MMASJCM_3480	3478464..3478601	+	45	hypothetical protein
MMASJCM_3481	3478630..3478947	-	105	hypothetical protein
MMASJCM_3482	3479161..3479442	+	93	hypothetical protein
MMASJCM_3484	3482249..3482464	+	71	hypothetical protein
MMASJCM_3485	3482481..3482795	-	104	hypothetical protein
MMASJCM_3486	3482974..3483234	+	86	hypothetical protein
MMASJCM_3487	3483748..3485670	-	640	hypothetical protein
MMASJCM_3488	3486239..3487597	-	452	hypothetical protein
MMASJCM_3489	3487929..3488300	-	123	hypothetical protein
MMASJCM_3490	3488293..3490251	-	652	hypothetical protein
MMASJCM_3491	3490377..3491396	-	339	hypothetical protein
MMASJCM_3492	3491495..3492070	-	191	hypothetical protein
MMASJCM_3493	3492067..3493542	-	491	putative phage integrase
MMASJCM_4522	4518649..4518885	+	78	hypothetical protein
MMASJCM_4523	4518882..4519169	+	95	hypothetical protein
MMASJCM_4524	4519198..4520571	+	457	hypothetical protein
MMASJCM_4525	4520604..4521251	+	215	hypothetical protein
