# Supplementary material for: Complete Genome Sequence and Comparative Genomic Analysis of Mycobacterium massiliense JCM 15300 in the Mycobacterium abscessus Group Reveal a Conserved Genomic Island MmGI-1 Related to Putative Lipid Metabolism
Source: PLoS One. 2014 Dec 11;9(12):e114848. doi: 10.1371/journal.pone.0114848 (PMC4263727; doi:10.1371/journal.pone.0114848)
Supplement: S3 Table — The deleted genes of massiliense and bolletii clusters among M. abscessus group. (PDF) [file pone.0114848.s007.pdf]

Table S3. The deleted genes of massiliense and bolletii clusters among *M. abscessus* group.

Gene_ID*	Location at M. abscessus ATCC 19977	Strand	Length	Product	Note
MAB_0059c	56726..57058	-	110	hypothetical protein	Del_M
MAB_0060	57232..57879	+	215	nitrate/nitrite response transcriptional regulatory protein NarL	Del_M
MAB_0061	57885..59072	+	395	two component sensor kinase	Del_M
MAB_0479	475516..475956	+	146	putative regulatory protein MarR	Del_M
MAB_0480	476024..477118	+	364	hypothetical protein	Del_M
MAB_0481c	477131..477499	-	122	hypothetical protein	Del_M
MAB_0482c	477496..478416	-	306	hypothetical protein	Del_M
MAB_0483c	478458..479093	-	211	TetR family transcriptional regulator	Del_M
MAB_0785	779916..780749	+	277	hypothetical protein	Del_M
MAB_0786	780620..781363	+	247	hypothetical protein	Del_M
MAB_0791	784406..784756	+	116	hypothetical protein	Del_M
MAB_0792	784753..785295	+	180	hypothetical protein	Del_M
MAB_0793	785292..785609	+	105	hypothetical protein	Del_M
MAB_0794	785606..786121	+	171	hypothetical protein	Del_M
MAB_0795	786164..786925	+	253	hypothetical protein	Del_M
MAB_0796c	786939..787736	-	265	hypothetical protein	Del_M
MAB_0797c	787744..788499	-	251	hypothetical protein	Del_M
MAB_0798c	788496..789029	-	177	hypothetical protein	Del_M
MAB_0799c	789026..789514	-	162	hypothetical protein	Del_M
MAB_0800	789820..790314	+	164	hypothetical protein	Del_M
MAB_0801	790400..791023	+	207	hypothetical protein	Del_M
MAB_0805	796263..796733	+	156	hypothetical protein	Del_M
MAB_0806	796742..797497	+	251	lipoprotein LppR	Del_M
MAB_0807	797731..798792	+	353	hypothetical protein	Del_M
MAB_0808c	799200..799592	-	130	hypothetical protein	Del_M
MAB_1043c	1052515..1053387	-	290	putative oxidoreductase	Del_M
MAB_1044c	1053384..1053878	-	164	glyoxalase/bleomycin resistance protein	Del_M
MAB_1045	1053960..1054520	+	186	hypothetical protein	Del_M
MAB_2255	2285340..2288708	+	1122	non-ribosomal peptide synthetase	Del_M
MAB_2256	2288717..2292490	+	1257	polyketide synthase	Del_M
MAB_2257	2292487..2297532	+	1681	polyketide synthase	Del_M
MAB_2258	2297529..2303486	+	1985	peptide synthetase and polyketide synthase	Del_M
MAB_2259	2303551..2304351	+	266	putative O-methyltransferase	Del_M
MAB_2260	2304472..2305410	+	312	putative formyltransferase	Del_M
MAB_3553	3598294..3599553	+	419	2-isopropylmalate synthase	Del_MB
MAB_3554	3599556..3600500	+	314	ketopantoate reductase ApbA/PanE	Del_MB
MAB_3555	3600497..3601900	+	467	major facilitator transporter	Del_MB
MAB_3556	3601910..3602815	+	301	alpha/beta fold hydrolase	Del_MB
MAB_3557	3602812..3603336	+	174	hypothetical protein	Del_MB
MAB_3558	3603323..3604441	+	372	hypothetical protein	Del_MB
MAB_3559c	3604412..3604846	-	144	hypothetical protein	Del_MB
MAB_3566c	3610719..3611699	-	326	putative cyclase	Del_MB
MAB_3567c	3611699..3612244	-	181	putative carboxymuconolactone decarboxylase	Del_MB
MAB_3568c	3612383..3613105	-	240	putative thioesterase	Del_MB
MAB_3569c	3613102..3614172	-	356	hypothetical protein	Del_MB
MAB_3570c	3614172..3614873	-	233	putative 4'-phosphopantetheinyl transferase	Del_MB
MAB_3571c	3614870..3615640	-	256	hypothetical protein	Del_MB
MAB_3572c	3615637..3615858	-	73	hypothetical protein	Del_MB
MAB_3573c	3615855..3617723	-	622	HAD family phosphatase FbkH	Del_MB
MAB_3574c	3617720..3618745	-	341	3-oxoacyl-[acyl-carrier-protein] synthase III	Del_MB
MAB_3575c	3618780..3621758	-	992	polyketide synthase	Del_MB
MAB_3576c	3621763..3622011	-	82	phosphopantetheine attachment site domain-containing protein	Del_MB
MAB_3577c	3622065..3623720	-	551	putative acyl-CoA dehydrogenase	Del_MB
MAB_3578c	3623717..3625450	-	577	acyl-CoA dehydrogenase	Del_MB
MAB_3579c	3625447..3627192	-	581	putative acyl-CoA synthase/polyketide synthase	Del_MB
MAB_4342c	4422631..4423068	-	145	putative MutT/nudix family protein	Del_MB
MAB_4343c	4423132..4423710	-	192	WhiB family transcriptional regulator	Del_MB
MAB_4344c	4423715..4427026	-	1103	putative NAD-dependent glutamate dehydrogenase	Del_MB
MAB_4347	4430039..4431640	+	533	putative regulatory protein	Del_MB
MAB_4349c	4432496..4433092	-	198	hypothetical protein	Del_MB
MAB_4350c	4433089..4434531	-	480	putative amino acid permease	Del_MB
MAB_4352c	4436178..4436777	-	199	TetR family transcriptional regulator	Del_MB
MAB_4353	4436811..4437263	+	150	hypothetical protein	Del_MB
MAB_4354	4437713..4438339	+	208	hypothetical protein	Del_MB
MAB_4356c	4439187..4442663	-	1158	indolepyruvate ferredoxin oxidoreductase	Del_MB
MAB_4357c	4442685..4444340	-	551	putative monooxygenase	Del_MB
MAB_4358c	4444337..4444828	-	163	putative biphenyl-2,3-diol 1,2-dioxygenase III	Del_MB
MAB_4360c	4445021..4445596	-	191	transcriptional regulator TetR	Del_MB
MAB_4361	4445695..4446561	+	288	fumarylacetoacetate hydrolase family protein	Del_MB
MAB_4362	4446558..4448414	+	618	putative long-chain-fatty-acid--CoA ligase	Del_MB
MAB_4802	4909957..4910622	+	221	hypothetical protein	Del_M
MAB_4803	4910648..4911025	+	125	hypothetical protein	Del_M

* *M. abscessus* ATCC 19977

Del_M: deleted CDS in massiliense cluster, Del_MB: deleted CDS in both massiliense and bolletii clusters.
