# Supplementary material for: Complete Genome Sequence and Comparative Genomic Analysis of Mycobacterium massiliense JCM 15300 in the Mycobacterium abscessus Group Reveal a Conserved Genomic Island MmGI-1 Related to Putative Lipid Metabolism
Source: PLoS One. 2014 Dec 11;9(12):e114848. doi: 10.1371/journal.pone.0114848 (PMC4263727; doi:10.1371/journal.pone.0114848)
Supplement: S4 Table — Oligonucleotide primer sequences used in PCR assays and the judging method for presence of MmGI-1 and other M. massiliense unique regions. (PDF) [file pone.0114848.s008.pdf]

Table S4. Oligonucleotide primer sequences used in PCR assays and the judging method for presence of MmGI-1 and other *M.massiliense* unique regions.

Region	Forward primer name	Sequence	Reverse primer name	Sequence	Amplified fragment length	PCR extension time*	Determination of positive or negative
MmGI-1	Mma-0986-F	5'-ctgcggccacagttcgtgtt-3'	Mma-0988-R	5'-acgggctgctgcaacacgtt-3'	1,225 bp	1 min	All amplicon positive: MmGI-1 positive<br>All amplicon negative: MmGI-1 negative
	Mma-1006-F	5'-tcgatggtgcgctgccactt-3'	Mma-1010-R	5'-acggcggctatcacaggctt-3'	3,063 bp	5 min	
	Mma-1014-F	5'-gggatgatagagaccgccga-3'	Mma-1016-R	5'-ccgcatagcaggcgttacga-3'	2,367 bp	5 min	
	Mma-1018-F	5'-gtcgaggctgccgaggtgtt-3'	Mma-1019-R	5'-tggcgcgatgccggtcgatt-3'	1,694 bp	5 min	
	Mma-1028-F	5'-gctccttgtccgcctgatgt-3'	Mma-1030-R	5'-cccgcgacaagacgttcgaa-3'	2,288 bp	5 min	
	Mma-1034-F	5'-gtcgaaggcgtcggtgacaa-3'	Mma-1038-R	5'-tcgcggacatggccaccaaa-3'	3,771 bp	5 min	
MMASJCM_2099..2100	Mma-2098-F	5'-gcgggtctcatcgcctgcta-3'	Mma-2101-R	5'-ggggtatcggccgcatgatt-3'	4,411 bp / 357 bp	5 min	4,411 bp amplicon: positive<br>357 bp amplicon: negative
MMASJCM-2507..2524	Mma-2509-F	5'-accgaacacgacgggcacaa-3'	Mma-2514-R	5'-tcgtggtcgtcgaccaggaa-3'	5,664 bp	5 min	5,664 bp amplicon: positive
	Mma-2505-F	5'-ccctccgttggcgtcgaaca-3'	Mma-2525-R	5'-gccccatgagcgatgaccaa-3'	565 bp	1 min	565 bp amplicon: negative
MMASJCM-4337..4346	Mma-4339-F	5'-ggcggcacgacaacgctcat-3'	Mma-4343-R	5'-agcgagcacgccatcaccat-3'	4,506 bp	5 min	4,506 bp amplicon: positive
	Mma-4335-F	5'-ctcatcctcgtcctcgccta-3'	Mma-4347-R	5'-accatcagccgcaccgcctt-3'	576 bp	1 min	576 bp amplicon: negative

* PCR was performed under the following conditions: at 98°C for 20 sec followed by 30 cycles at 98°C for 15 sec, 65°C for 15 sec and 68°C for 1 min (for below 1.5 kb amplicons) or 5 min (for over 1.5 kb amplicons). See also Materials and Methods.
