# Supplementary material for: Complete Genome Sequence and Comparative Genomic Analysis of Mycobacterium massiliense JCM 15300 in the Mycobacterium abscessus Group Reveal a Conserved Genomic Island MmGI-1 Related to Putative Lipid Metabolism
Source: PLoS One. 2014 Dec 11;9(12):e114848. doi: 10.1371/journal.pone.0114848 (PMC4263727; doi:10.1371/journal.pone.0114848)
Supplement: S5 Table — Isolates analyzed in the present study and results of conventional PCR based detection against MmGI-1 and other M. massiliense unique regions. (PDF) [file pone.0114848.s009.pdf]

Table S5. Isolates analyzed in the present study and results of conventional PCR based detection against MmGI-1 and other *M.massiliense* unique regions.

strain name	Organism	colony morphology	Isolated from	Place	Year	Results of detected region by conventional PCR method				Culture experiment
						MmGI-1 (see Fig. 2)	MMASJCM-2099..2100 (see Fig. 3A)	MMASJCM-2507..2524 (see Fig. 3B)	MMASJCM-4337..4346 (see Fig. 3C)	
LRC-Abs3	*M. abscessus*	rough type	Respiratory system	Hokkaido	2001	-	-	-	-	N.E.
LRC-ABs7	*M. abscessus*	rough type	Respiratory system	Hokkaido	2005	-	-	-	-	N.E.
LRC-Abs64	*M. abscessus*	rough type	Respiratory system	Osaka	2006	-	-	-	-	N.E.
LRC-Abs80	*M. abscessus*	rough type	Respiratory system	Fukuoka	2006	-	-	-	-	N.E.
LRC-Abs14	*M. abscessus*	rough type	Respiratory system	Hiroshima	2007	-	-	-	-	N.E.
LRC-Abs37	*M. abscessus*	rough type	Respiratory system	Tokyo	2007	-	-	-	-	N.E.
LRC-Abs99	*M. abscessus*	rough type	Respiratory system	Tokyo	2007	-	-	-	-	N.E.
LRC-Abs15	*M. abscessus*	rough type	Respiratory system	Hiroshima	2008	-	-	-	-	N.E.
LRC-Abs16	*M. abscessus*	rough type	Respiratory system	Hiroshima	2008	-	-	-	-	N.E.
LRC-Abs17	*M. abscessus*	rough type	Respiratory system	Hiroshima	2008	-	-	-	-	N.E.
LRC-Abs42	*M. abscessus*	rough type	Respiratory system	Tokyo	2008	-	-	-	-	N.E.
LRC-Abs45	*M. abscessus*	rough type	Respiratory system	Tokyo	2008	-	-	-	-	N.E.
LRC-Abs46	*M. abscessus*	rough type	Respiratory system	Tokyo	2008	-	-	-	-	N.E.
LRC-Abs47	*M. abscessus*	rough type	Respiratory system	Tokyo	2008	-	-	-	-	N.E.
LRC-Abs48	*M. abscessus*	rough type	Respiratory system	Tokyo	2008	-	-	-	-	N.E.
LRC-Abs87	*M. abscessus*	rough type	Respiratory system	Tokyo	2008	-	-	-	-	N.E.
LRC-Abs90	*M. abscessus*	rough type	Respiratory system	Tokyo	2008	-	-	-	-	N.E.
LRC-Abs105	*M. abscessus*	rough type	Respiratory system	Tokyo	2009	-	-	-	-	N.E.
LRC-Abs13	*M. abscessus*	rough type	Respiratory system	Osaka	2009	-	-	-	-	N.E.
LRC-Abs21	*M. abscessus*	rough type	Respiratory system	Osaka	2009	-	-	-	-	N.E.
LRC-Abs22	*M. abscessus*	rough type	Respiratory system	Osaka	2009	-	-	-	-	N.E.
LRC-Abs24	*M. abscessus*	rough type	Respiratory system	Osaka	2009	-	-	-	-	N.E.
LRC-Abs35	*M. abscessus*	rough type	Respiratory system	Osaka	2009	-	-	-	-	N.E.
LRC-Abs36	*M. abscessus*	rough type	Respiratory system	Osaka	2009	-	-	-	-	N.E.
LRC-Abs84	*M. abscessus*	rough type	Respiratory system	Kanagawa	2010	-	-	-	-	N.E.
LRC-Abs85	*M. abscessus*	rough type	Respiratory system	Kanagawa	2010	-	-	-	-	N.E.
LRC-AbsA12	*M. abscessus*	rough type	Skin lesion	Kanagawa	2010	-	-	-	-	N.E.
LRC-AbsA15	*M. abscessus*	rough type	Skin lesion	Tokyo	2011	-	-	-	-	N.E.
LRC-Abs52	*M. abscessus*	smooth type	Respiratory system	Osaka	2000	-	-	-	-	N.E.
LRC-Abs56	*M. abscessus*	smooth type	Respiratory system	Osaka	2001	-	-	-	-	N.E.
LRC-Abs58	*M. abscessus*	smooth type	Respiratory system	Osaka	2001	-	-	-	-	N.E.
LRC-Abs60	*M. abscessus*	smooth type	Respiratory system	Osaka	2002	-	-	-	-	N.E.
LRC-Abs79	*M. abscessus*	smooth type	Respiratory system	Fukuoka	2003	-	-	-	-	N.E.
LRC-Abs100	*M. abscessus*	smooth type	Respiratory system	Tokyo	2005	-	-	-	-	in this study
LRC-Abs62	*M. abscessus*	smooth type	Respiratory system	Osaka	2005	-	-	-	-	N.E.
LRC-Abs95	*M. abscessus*	smooth type	Respiratory system	Tokyo	2006	-	-	-	-	N.E.
LRC-Abs96	*M. abscessus*	smooth type	Respiratory system	Tokyo	2006	-	-	-	-	N.E.
LRC-Abs97	*M. abscessus*	smooth type	Respiratory system	Tokyo	2006	-	-	-	-	N.E.
LRC-Abs39	*M. abscessus*	smooth type	Respiratory system	Tokyo	2007	-	-	-	-	N.E.
LRC-Abs40	*M. abscessus*	smooth type	Respiratory system	Tokyo	2007	-	-	-	-	in this study
LRC-Abs10	*M. abscessus*	smooth type	Respiratory system	Hokkaido	2008	-	-	-	-	N.E.
LRC-Abs41	*M. abscessus*	smooth type	Respiratory system	Tokyo	2008	-	-	-	-	N.E.
LRC-Abs43	*M. abscessus*	smooth type	Respiratory system	Tokyo	2008	-	-	-	-	N.E.
LRC-Abs69	*M. abscessus*	smooth type	Respiratory system	Osaka	2008	-	-	-	-	N.E.
LRC-Abs71	*M. abscessus*	smooth type	Respiratory system	Shimane	2008	-	-	-	-	N.E.
LRC-Abs72	*M. abscessus*	smooth type	Respiratory system	Shimane	2008	-	-	-	-	N.E.
LRC-Abs101	*M. abscessus*	smooth type	Respiratory system	Tokyo	2009	-	-	-	-	N.E.
LRC-Abs29	*M. abscessus*	smooth type	Respiratory system	Osaka	2009	-	-	-	-	N.E.
LRC-Abs76	*M. abscessus*	smooth type	Respiratory system	Hiroshima	2009	-	-	-	-	N.E.
LRC-Abs83	*M. abscessus*	smooth type	Respiratory system	Kyoto	2009	-	-	-	-	N.E.
LRC-Abs106	*M. abscessus*	smooth type	Respiratory system	Wakayama	2010	-	-	-	-	in this study
LRC-Abs110	*M. abscessus*	smooth type	Respiratory system	Hokkaido	2010	-	-	-	-	in this study
ATCC19977	*M. abscessus*	smooth type	Skin lesion	N.A.	1953	-	-	-	-	in this study
LRC-Abs53	*M. abscessus*	rough type	Respiratory system	Osaka	2000	-	-	+	-	N.E.
LRC-Abs66	*M. abscessus*	rough type	Respiratory system	Osaka	2007	-	-	+	-	N.E.
LRC-Abs89	*M. abscessus*	rough type	Respiratory system	Tokyo	2008	-	-	+	-	N.E.
LRC-Abs18	*M. abscessus*	rough type	Respiratory system	Hiroshima	2009	-	-	+	-	N.E.
LRC-Abs93	*M. abscessus*	rough type	Respiratory system	Tokyo	2009	-	-	+	-	N.E.
LRC-Abs59	*M. abscessus*	smooth type	Respiratory system	Osaka	2002	-	-	+	-	N.E.
LRC-Abs61	*M. abscessus*	smooth type	Respiratory system	Osaka	2003	-	-	+	-	in this study
LRC-Abs38	*M. abscessus*	smooth type	Respiratory system	Tokyo	2007	-	-	+	-	in this study
LRC-Abs44	*M. abscessus*	smooth type	Respiratory system	Tokyo	2008	-	-	+	-	in this study
LRC-Abs1	*M. abscessus*	smooth type	Respiratory system	Kanagawa	2009	-	-	+	-	N.E.
LRC-Abs12	*M. abscessus*	smooth type	Respiratory system	Osaka	2009	-	-	+	-	in this study
LRC-Abs70	*M. abscessus*	rough type	Respiratory system	Shimane	2008	-	-	-	+	N.E.
LRC-Abs67	*M. massiliense*	smooth type	Respiratory system	Osaka	2007	-	-	-	+	N.E.
LRC-Abs11	*M. abscessus*	smooth type	Respiratory system	Hokkaido	2008	-	-	-	+	in this study
LRC-Abs51	*M. abscessus*	smooth type	Respiratory system	Tokyo	2009	-	-	-	+	in this study
LRC-Abs9	*M. abscessus*	rough type	Respiratory system	Hokkaido	2007	-	-	+	+	N.E.
LRC-Abs20	*M. massiliense*	smooth type	Respiratory system	Okayama	2009	-	-	+	+	in this study
LRC-Abs73	*M. massiliense*	smooth type	Respiratory system	Hiroshima	2009	-	-	+	+	in this study
LRC-Abs75	*M. abscessus*	smooth type	Respiratory system	Hiroshima	2009	-	-	+	+	in this study
LRC-Abs107	*M. massiliense*	smooth type	Respiratory system	Okayama	2010	-	-	+	+	in this study
LRC-Abs92	*M. abscessus*	rough type	Respiratory system	Tokyo	2007	+	-	+	-	N.E.
LRC-Abs55	*M. massiliense*	rough type	Respiratory system	Osaka	2000	-	+	-	-	N.E.
LRC-Abs77	*M. massiliense*	rough type	Respiratory system	Fukuoka	2004	-	+	-	-	N.E.
LRC-Abs94	*M. massiliense*	rough type	Respiratory system	Tokyo	2006	-	+	-	-	N.E.
LRC-Abs91	*M. massiliense*	rough type	Respiratory system	Tokyo	2008	-	+	-	-	N.E.
LRC-Abs65	*M. massiliense*	rough type	Respiratory system	Osaka	2006	-	+	+	-	N.E.
LRC-Abs50	*M. massiliense*	rough type	Respiratory system	Tokyo	2009	-	+	+	-	N.E.
LRC-Abs88	*M. massiliense*	rough type	Respiratory system	Tokyo	2008	-	+	-	+	N.E.
LRC-Abs74	*M. massiliense*	rough type	Respiratory system	Hiroshima	2009	-	+	-	+	N.E.
LRC-Abs2	*M. massiliense*	rough type	Respiratory system	Hokkaido	2000	-	+	+	+	N.E.
LRC-Abs4	*M. massiliense*	rough type	Respiratory system	Hokkaido	2001	-	+	+	+	N.E.
LRC-Abs5	*M. massiliense*	rough type	Respiratory system	Hokkaido	2002	-	+	+	+	N.E.
LRC-Abs102	*M. massiliense*	rough type	Respiratory system	Tokyo	2008	-	+	+	+	N.E.

LRC-Abs98	*M. massiliense*	rough type	Respiratory system	Tokyo	2008	-	+	+	+	N.E.
LRC-AbsA2	*M. massiliense*	rough type	Skin lesion	Shizuoka	2008	-	+	+	+	N.E.
LRC-Abs25	*M. massiliense*	rough type	Respiratory system	Osaka	2009	-	+	+	+	N.E.
LRC-Abs34	*M. massiliense*	rough type	Respiratory system	Osaka	2009	-	+	+	+	N.E.
LRC-Abs82	*M. massiliense*	rough type	Skin lesion	Tokyo	2009	-	+	+	+	N.E.
LRC-AbsA10	*M. massiliense*	rough type	Skin lesion	Tokyo	2009	-	+	+	+	N.E.
LRC-AbsA13	*M. massiliense*	rough type	Skin lesion	Saitama	2010	-	+	+	+	N.E.
LRC-AbsA14	*M. massiliense*	rough type	Skin lesion	Saitama	2010	-	+	+	+	N.E.
LRC-Abs104	*M. massiliense*	smooth type	Respiratory system	Tokyo	2008	-	+	+	+	in this study
LRC-AbsA1	*M. massiliense*	smooth type	Skin lesion	Shizuoka	2008	-	+	+	+	in this study
LRC-AbsB1	*M. massiliense*	smooth type	Environment	Shizuoka	2008	-	+	+	+	in this study
LRC-Abs19	*M. massiliense*	smooth type	Respiratory system	Okayama	2009	-	+	+	+	in this study
LRC-Abs112	*M. massiliense*	smooth type	Blood	Saitama	2010	-	+	+	+	in this study
LRC-AbsA11	*M. massiliense*	smooth type	Skin lesion	Saitama	2010	-	+	+	+	N.E.
LRC-Abs103	*M. massiliense*	rough type	Respiratory system	Tokyo	2007	+	+	-	-	N.E.
LRC-Abs30	*M. massiliense*	rough type	Respiratory system	Osaka	2009	+	+	-	-	N.E.
LRC-Abs109	*M. massiliense*	smooth type	Respiratory system	Hokkaido	2009	+	+	-	-	in this study
LRC-Abs54	*M. massiliense*	rough type	Respiratory system	Osaka	2000	+	+	+	-	N.E.
LRC-Abs8	*M. massiliense*	rough type	Respiratory system	Hokkaido	2006	+	+	+	-	N.E.
LRC-Abs23	*M. massiliense*	rough type	Respiratory system	Osaka	2009	+	+	+	-	N.E.
LRC-Abs57	*M. massiliense*	smooth type	Respiratory system	Osaka	2001	+	+	+	-	in this study
LRC-Abs6	*M. massiliense*	smooth type	Respiratory system	Hokkaido	2003	+	+	+	-	in this study
LRC-Abs63	*M. massiliense*	smooth type	Respiratory system	Osaka	2006	+	+	+	-	in this study
LRC-Abs68	*M. massiliense*	smooth type	Respiratory system	Osaka	2008	+	+	+	-	in this study
LRC-Abs31	*M. massiliense*	smooth type	Respiratory system	Osaka	2009	+	+	+	-	N.E.
LRC-Abs33	*M. massiliense*	smooth type	Respiratory system	Osaka	2009	+	+	+	-	N.E.
LRC-Abs108	*M. massiliense*	smooth type	Respiratory system	Hokkaido	2009	+	+	+	+	in this study
JCM15300	*M. massiliense*	smooth type	Respiratory system	France	N.A.	+	+	+	+	in this study

N.A.: Not appicable, N.E.: Not examined
